# Multi-tissue analysis identifies mitochondrial genes in chicken aging-induced productivity decline

**DOI:** 10.1186/s40104-026-01392-0

**Published:** 2026-04-22

**Authors:** Mingyue Gao, Junnan Zhang, Boxuan Zhang, Xinwei Jiang, Bowen Niu, Conghao Zhong, Fangren Lan, Wenxin Zhang, Ning Yang, Congjiao Sun

**Affiliations:** 1 State Key Laboratory of Animal Biotech Breeding, Frontier Science Center of Molecular Design Breeding, China Agricultural University, Beijing, 100193 China; 2 National Engineering Laboratory for Animal Breeding, College of Animal Science and Technology, China Agricultural University, Beijing, 100193 China

**Keywords:** Age-related genes, Chicken, Functional decline, Mitochondrial dysfunction, Multi-tissue RNA-seq

## Abstract

**Background:**

Aging-related production decline is widely observed in poultry farming, making it challenging to prolong the laying cycle of hens. However, its genetic determinants remain unclear. This study aimed to systematically identify key genes associated with hens with age-related production decline and reveal dynamic changes in gene expression during tissue functional decline.

**Results:**

This study integrated multi-tissue transcriptomic data from 216 samples obtained from Rhode Island Red laying hens, covering nine tissues (hypothalamus, pituitary, cecum, duodenum, liver, ovary, magnum, isthmus, and uterus) at three age points (50, 70, and 100 weeks of age). Through comprehensive analysis, we identified 87 genes that showed significant changes across all nine tissues (|log_2_-fold change| > 1, FDR < 0.05) and 20 hub genes playing critical roles in mitochondrial and ribosome functions. In addition, we identified 106 differentially expressed genes (DEGs) with tissue-specific expression patterns and interaction networks. We further screened 51 genes that are associated with both the age-related production decline process and economically important traits in chickens. Finally, functional validation using a hydrogen peroxide-induced senescence model in DF-1 cells highlighted *NDUFB9*, a gene associated with mitochondrial respiratory chain complex I, as a potential anti-aging target.

**Conclusions:**

This study provides a systematic multi-tissue transcriptomic framework for understanding aging-associated productivity decline in laying hens. Our findings highlight conserved mitochondrial and ribosomal dysfunction as key molecular features of age-related production decline and identify *NDUFB9* as a potential anti-aging target, offering valuable insights for improving poultry longevity and production performance.

**Graphical Abstract:**

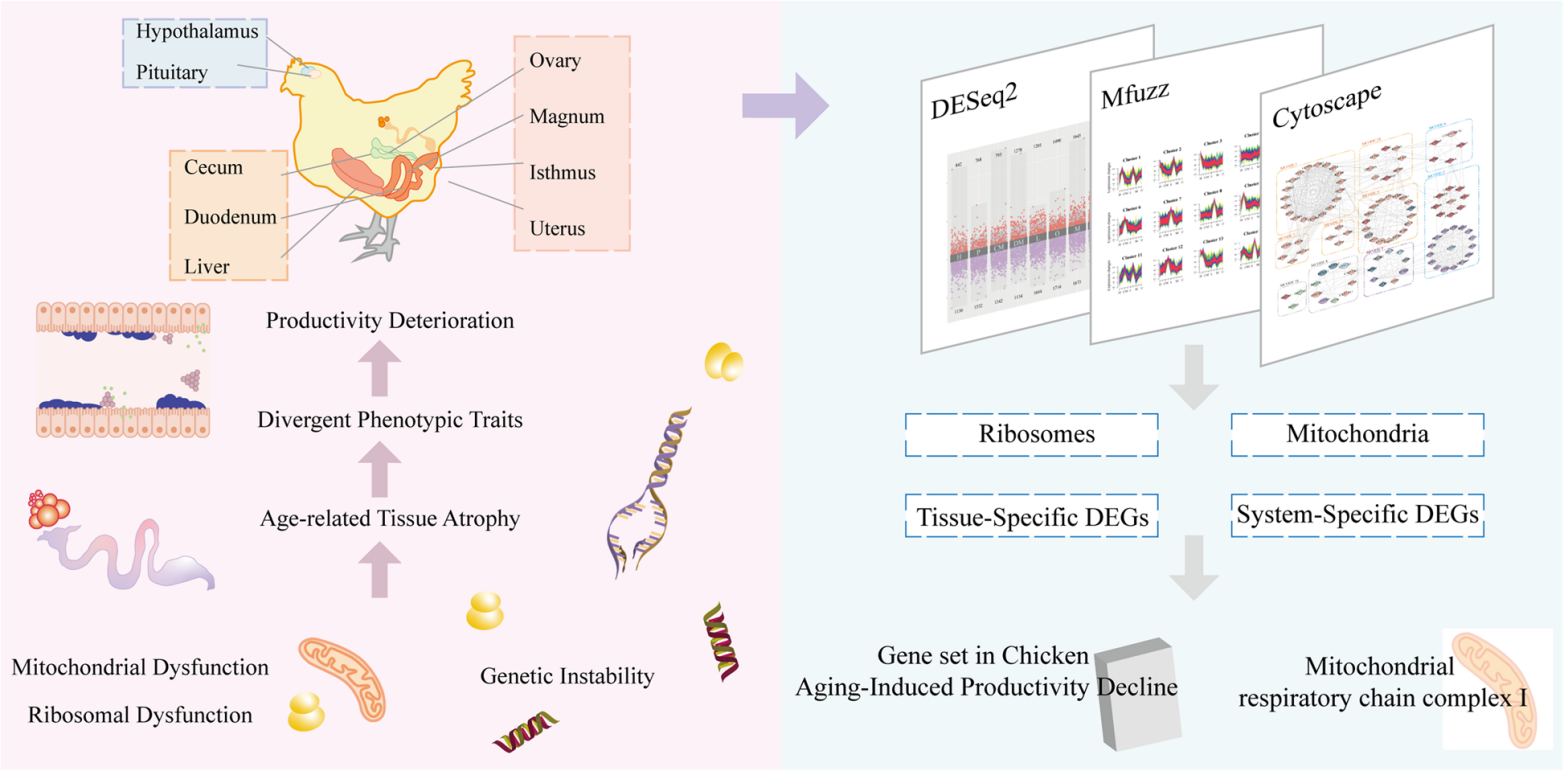

**Supplementary Information:**

The online version contains supplementary material available at 10.1186/s40104-026-01392-0.

## Introduction

Aging research has explored the functional decline of organisms during adulthood [1], which is a fundamental biological process marked by general deterioration in tissue function and decreased resistance to inflammation and infection [2]. Tissue functional deterioration during the aging process has been comprehensively characterized by López-Otín et al. [1], who summarized twelve hallmark features of aging, such as genomic instability, telomere attrition, and related molecular and cellular alterations. Additionally, scientists have compiled a gene set (SenMayo) and an aging database (CellAge) to facilitate human aging-related research [3, 4]. Some studies have even suggested that aging may be a malleable and potentially reversible process [5]. Importantly, aging-related molecular alterations often exert pronounced effects on highly dynamic and hormone-regulated tissues, including the reproductive system.

During the production cycle of laying hens, reproductive function transitions from maturation to decline, resulting in an initial increase and subsequent decrease in egg-laying performance. Notably, this suggests that functional deterioration of reproductive tissues emerges during the late laying period, which corresponds to an early stage of the hens’ overall lifespan. In recent years, driven by rising feed costs and advances in poultry management and breeding strategies, the feeding cycle of laying hens has been progressively extended to improve economic efficiency. The current breeding objective is to produce 500 high-quality eggs within 100 weeks of age [6]. However, the extension of the breeding cycle can result in a higher proportion of older laying hens, which leads to a decline in both the efficiency and quality of egg production [7–10]. Together, these age-associated changes suggest that reproductive aging is a key limiting factor in extended-cycle laying hens. Therefore, from a genetic and molecular perspective, elucidating the regulatory mechanisms underlying aging-related changes in reproductive tissues is essential for understanding the biological basis of egg production decline and for developing strategies to improve productivity and egg quality in older laying hens.

Research indicates that the decline in production performance among laying hens is primarily attributable to increased heterogeneity. Consequently, some individuals possess the ability to sustain high production over extended periods, suggesting that extending the production lifespan and maintaining elevated production performance in chickens is feasible [6]. Existing studies on hens with age-related production decline have primarily focused on specific traits or individual tissues. For instance, Li et al. [11] elucidated the relationship between changes in the ionome and the age-related production decline process in chickens, offering insights into the regulation of aging in chickens. Aging inevitably leads to wear and tear of the reproductive system of chicken, resulting in diminished efficiency and a decline in egg production [12, 13]. Additionally, some researchers have studied the effects of aging on egg quality [14]. Despite these advances, most previous studies have been limited to single tissues or tissues within the same physiological system, thereby failing to capture aging as a coordinated, organism-wide process. A systematic, multi-tissue investigation of aging-related molecular changes across the body of laying hens remains lacking, hindering a comprehensive understanding of how aging influences reproductive performance and production traits at the whole-organism level.

In summary, analysis of aging is becoming increasingly prevalent; however, the genetic regulatory mechanisms underlying tissue function decline in chickens remain poorly understood, and the specific gene set associated with aging-induced productivity decline in chickens is yet to be identified. In this study, we aimed to systematically characterize aging-associated molecular changes across the laying cycle by integrating transcriptomic profiles from nine tissues collected at multiple age points. By constructing tissue-specific functional decline maps and tissue interaction networks, we sought to identify key regulatory genes and pathways associated with reproductive aging and productivity decline in chickens. The multi-tissue, longitudinal design of this study provides a comprehensive framework for understanding the molecular basis of aging-related functional deterioration and offers potential targets for mitigating productivity loss and informing molecular breeding strategies in laying hens.

## Materials and methods

### Experiment animals and sample collection

The Rhode Island Red chickens used in this study were maintained under uniform husbandry conditions in individual cages throughout the experimental period. To capture representative biological changes associated with aging, chickens with trait values closest to the group mean were selected for tissue collection at 50 (*n* = 6), 70 (*n* = 6), and 100 (*n* = 12) weeks of age. Multiple tissues were collected from each chicken, including hypothalamus, pituitary, cecum, duodenum, liver, ovary, magnum, isthmus, and uterus, resulting in a total of 216 samples for downstream analyses. Additionally, to validate the age-related production decline findings, RNA-seq data from an independent cohort of hens (*n* = 5) that had undergone a standardized forced molting regimen (induced by a high-zinc diet at 80 weeks of age) were incorporated into the analysis. These hens were sampled at 100 weeks of age, well after molting completion and the resumption of stable egg production.

### Phenotype analysis of different traits

The number of eggs laid by each individual was recorded at 49, 50, 69, 70, 97, and 98 weeks of age. Egg-laying rate (ELR) for 50, 70, and 100 weeks were calculated based on the average egg counts from 49–50 weeks, 69–70 weeks, and 97–98 weeks, respectively. Egg quality traits were measured over three consecutive days at each time point, and the mean values were used as phenotypic measurements. The assessed traits included eggshell strength (ESS) and Haugh unit (HU). To evaluate the effects of age on ELR, ESS, and HU, analysis of variance (ANOVA) was conducted using the aov function in R.

### RNA extraction, quality assessment, library preparation, and sequencing

Total RNA was extracted from tissue samples using TRIzol reagent (Thermo Fisher Scientific, Waltham, MA, USA; Cat. No. 15596018) according to the manufacturer’s protocol. Briefly, tissues were homogenized in TRIzol reagent, followed by phase separation and RNA purification. RNA concentration and purity were assessed using a NanoDrop ND-1000 spectrophotometer (NanoDrop Technologies, Wilmington, DE, USA), and RNA integrity was evaluated using an Agilent 2100 Bioanalyzer (Agilent Technologies, CA, USA). Only samples with RNA concentrations > 50 ng/μL, RNA integrity number (RIN) > 7.0, and total RNA amount > 1 μg were used for downstream library construction.

Poly(A) + mRNA was enriched from total RNA through two rounds of purification using oligo(dT) magnetic beads (Dynabeads Oligo(dT), Thermo Fisher Scientific, Cat. No. 25–61005). Purified mRNA was fragmented at 94 °C for 5 min using magnesium-based fragmentation buffer (NEBNext^®^ Magnesium RNA Fragmentation Module, New England Biolabs, Cat. No. E6150S). First-strand cDNA synthesis was performed using SuperScript™ II Reverse Transcriptase (Invitrogen, Cat. No. 1896649), followed by second-strand cDNA synthesis using *E. coli* DNA Polymerase I (NEB, Cat. No. M0209) and RNase H (NEB, Cat. No. M0297), with incorporation of dUTP (Thermo Fisher Scientific, Cat. No. R0133) to enable strand-specific library construction.

The resulting double-stranded cDNA fragments were end-repaired, A-tailed, adapter-ligated, and size-selected using magnetic beads to generate libraries with an insert size of approximately 300 ± 50 bp. Libraries were subsequently amplified by PCR using a high-fidelity DNA polymerase (14 cycles) to selectively enrich non–dUTP-containing strands, producing strand-specific libraries. Final libraries were sequenced on an Illumina NovaSeq 6000 platform (LC-Bio Technology Co., Ltd., Hangzhou, China) using paired-end sequencing with a read length of 150 bp (PE150).

### Transcriptome data processing

#### Quality control

Raw reads were processed using Cutadapt (v1.9) to obtain clean reads by removing reads containing adapter sequences, polyA or polyG sequences, more than 5% unknown nucleotides (N), or reads failing predefined base-quality criteria (i.e., more than 20% of bases with a Phred quality score ≤ 20). After filtering, the quality of the resulting clean reads was assessed using FastQC (v0.11.9), including evaluation of Q20, Q30, and GC content [15, 16].

#### Reads alignment

Firstly, we constructed an index for the chicken genome (*Gallus gallus*, GCA 016699485.1, https://mart.ensembl.org/Gallus_gallus/Info/Annotation#) using STAR (v2.5.1), incorporating gene annotation information (–sjdbGTFfile) with a splice junction database overhang of 149 bp, corresponding to the read length of PE150 sequencing. Clean paired-end reads were then aligned to the reference genome using STAR with default parameters for paired-end RNA-seq data. Key parameters included on-the-fly decompression of gzipped FASTQ files, multithreaded alignment, and generation of coordinate-sorted BAM files (–outSAMtype BAM SortedByCoordinate).

#### Transcript assembly

Gene-level expression was quantified using StringTie (v1.3.1c) based on reads aligned to the chicken reference genome [17–19]. We provided the reference genome annotation (GTF) to StringTie and used the -e and -B options to estimate expression only for annotated genes and prepare outputs for downstream analysis. Gene-level normalized expression values were calculated using the Transcripts Per Kilobase of exon model per million mapped reads (TPM) method, and raw counts were obtained for each sample. Counts across samples were then merged using prepDE.py to generate matrices for differential expression analysis. By using this strategy, we focused exclusively on protein-coding genes annotated in the chicken genome, ensuring that downstream differential expression analysis reflects well-annotated genes only.

#### Principal component analysis

Principal component analysis (PCA) was performed on normalized TPM expression data using the prcomp function in R (v4.2.3). Prior to PCA, genes with zero variance or missing values were removed, and data were log-transformed. The resulting principal components were visualized using the factoextra (v1.0.7) and ggfortify (v0.4.17) R packages, with 95% confidence ellipses added for group separation.

#### Differential expression analysis

Differential expression analysis was performed using the DESeq2 package (v1.38.3) in R based on raw counts data [20]. Prior to analysis, genes were filtered based on their expression trends across the three age points (50, 70, and 100 weeks), and only those showing consistent up- or down-regulation were retained for differential analysis. The resulting *P* values were adjusted using the Benjamini and Hochberg method to control the false discovery rate (FDR) [21], and transcripts with FDR < 0.05 and |log_2_-fold change| > 1 were identified as DEGs.

#### Gene Ontology and KEGG pathway enrichment analysis

Functional enrichment analysis was conducted to identify the biological pathways associated with the aging-related DEGs. The GO database (http://www.geneontology.org/) categorizes functions into three domains: molecular function, biological process, and cellular component [22] using the ClusterProfiler package (v4.6.2) in R. The KEGG database (http://www.genome.jp/kegg/) provides insights into biological systems from systemic functional, genomic, and chemical perspectives [23]. For enrichment analysis, DEGs were first converted from gene symbols to Entrez IDs using the bitr function from the org.Gg.eg.db annotation database. The background gene set was defined as all genes detected above the expression threshold in our dataset, to minimize bias from unexpressed genes. Enrichment was tested using the hypergeometric test (one-sided) implemented in enrichGO and enrichKEGG, with *P* values adjusted by the Benjamini–Hochberg (BH) method to control for FDR. GO terms and KEGG pathways with adjusted *q* values < 0.05 were considered significantly enriched [24, 25].

#### Visualization analysis

The above analysis results were visualized using ggplot2 (v3.5.1).

### Gene set enrichment analysis

We employed gene set enrichment analysis (GSEA) using the clusterProfiler package (v4.6.2) in R to evaluate pathway enrichment of aging-associated differentially expressed genes across multiple tissues. KEGG and GO enrichment analyses were conducted using the gseKEGG and gseGO functions, respectively. GSEA visualization was performed using the enrichplot package (v1.18.4), and gene annotation was based on the org.Gg.eg.db database (v3.16.0). Pathways with |NES| > 1, *P* value < 0.05, and adjusted *P* value (*P*.adj) < 0.25 were considered significantly enriched [26].

### Soft clustering analysis

Soft clustering enables the assignment of a gene to multiple clusters by applying the fuzzy c-means algorithm to time-course gene expression data [27]. To identify tissue-specific genes that exhibited high expression levels in particular tissues at various age points, we employed the Mfuzz R package (v2.58.0) to perform a soft clustering enrichment analysis of gene expressions across different tissues at three distinct ages (50, 70, and 100 weeks). To minimize the impact of a gene being represented in multiple clusters, we extracted genes with MEM.SHIP > 0.5 across different clusters as the clustering results of Mfuzz. This approach yielded tissue-specific genes at various age points, facilitating the analysis of specific changes occurring in tissues throughout the aging process.

### PPI network construction and network integration

A protein–protein interaction (PPI) network of DEGs was constructed using the Search Tool for the Retrieval of Interacting Genes/Proteins (STRING) database [28]. PPI pairs with a combined score exceeding 0.4, which indicated the required confidence, were selected to construct the PPI network using Cytoscape software (v3.10.3) [29]. The Molecular Complex Detection (MCODE) plug-in in Cytoscape was employed to identify modules within the PPI network. The inferred modules were generated using default settings, with a degree cutoff of 2, node score cutoff of 0.2, K-core of 2, and maximum depth of 100. Furthermore, the CytoHubba application in Cytoscape was used to explore hub genes within the interactome network through several topological algorithms and various centralities based on the shortest paths [30]. We identified the top 10 ranked nodes using the NCC method. These algorithms highlight hub genes that encode core proteins, which may serve as key candidate genes with essential biological regulatory functions.

### Construction of gene networks in system-specific pathways

The samples were categorized into three systems: the reproductive system (ovary, magnum, isthmus, and uterus), digestive system (cecum, duodenum, liver), and nervous system (hypothalamus, pituitary). Base on system grouping, we identified DEGs unique to different systems, which we designated as system-specific DEGs. We then conducted functional enrichment analysis of these system-specific DEGs, focusing on the calcium signaling pathway and the neuroactive ligand-receptor interaction pathway.

### RNA extraction and reverse transcription

Total RNA was extracted from DF-1 cells using Trizol reagent (Takara Bio Inc., Kusatsu, Japan) according to the manufacturer's instructions. RNA was then reverse-transcribed using the PrimeScript RT reagent kit with gDNA Eraser (Takara Bio Inc., Kusatsu, Japan), and the reverse transcription product was used for amplification to obtain cDNA from DF-1 cells.

### Construction of overexpression plasmids

We designed primers using SnapGene (v8.0.1) and DNAMAN (v7) software and amplified the target fragment from the cDNA obtained from DF-1 cells using the KOD FX Neo high-fidelity enzyme (TOYOBO, Osaka, Japan; Table S17). The target fragment was then inserted into the pPB-EF1a-EGFP-puro plasmid using the NheI restriction enzyme site to construct an overexpression plasmid for the corresponding gene. The nucleotide sequences of the constructed plasmids were confirmed by sequencing analysis (RuiBiotech, Beijing, China).

### Cell culture and transfection

DF-1 cells were cultured in DMEM F12 medium with GlutaMAX supplement (Thermo Fisher, Beijing, China) and fetal bovine serum, with antibiotics added to the culture medium. Transfection was performed using Opti-MEM medium as a solvent and FuGENE as a transfection reagent, following the recommended system in the instructions. After 48 h, the cells were selected for Puromycin to eliminate non-transduced cells, and the normal culture medium was replaced 24 h later. At this point, surviving cells were successfully transfected with the overexpression plasmid.

### Cell treatments

To induce aging, DF-1 cells that had been passaged 15 times were treated with 10, 20, 50, 100, and 200 μmol/L hydrogen peroxide for 24 h, and then stained using the β-galactosidase staining kit and mitochondrial membrane potential and apoptosis detection kit with MitoTracker Red CMXRos and Annexin V-FITC (Beyotime, Shanghai, China).

### SA-β-Gal staining

After the intervention, cells were fixed by SA-β-Gal staining fixative for 15 min. An appropriate amount of staining solution (Beyotime, Shanghai, China) was prepared and added to the wells of the plate after washing with PBS. The plate was then placed in a 37 °C incubator (a CO_2_ incubator was not used) for 12 h. Finally, cells were visualized under a light microscope.

### Mitochondrial membrane potential and apoptosis detection using Mito-Tracker Red CMXRos and Annexin V-FITC

DF-1 cells treated with a hydrogen peroxide gradient and untreated DF-1 cells were washed with PBS in a 12-well plate. Following the instructions of the mitochondrial membrane potential and apoptosis detection kit with MitoTracker Red CMXRos and Annexin V-FITC, 188 μL of Annexin V-FITC binding solution, 5 μL of Annexin V-FITC, 2 μL of MitoTracker Red CMXRos staining solution, and 5 μL of Hoechst 33342 staining solution were sequentially added (Beyotime, Shanghai, China). After incubation at room temperature in the dark for 30 min, the cells were observed and photographed under a fluorescence microscope.

## Results

### Phenotype and RNA-seq data quality assessment in samples

**Fig. 1:**
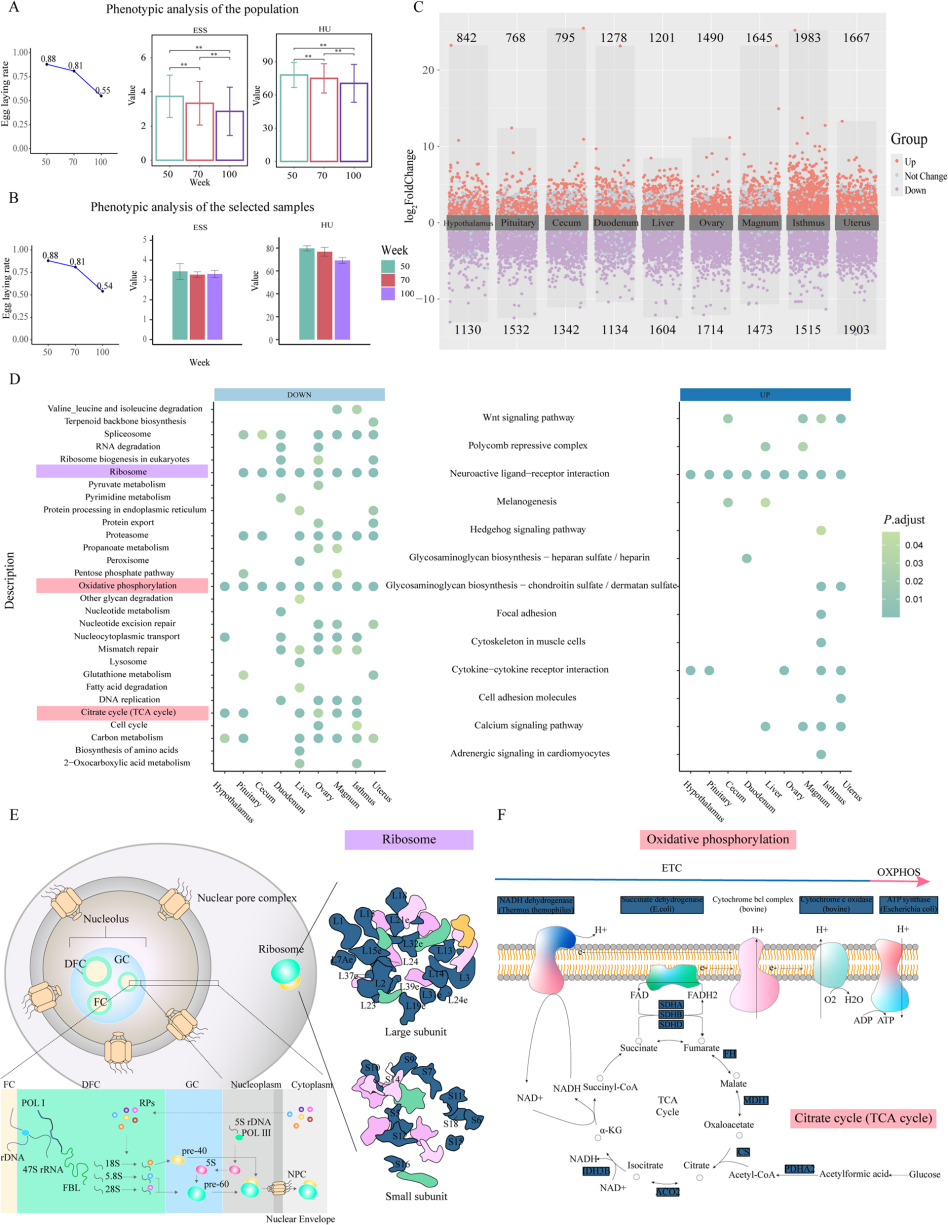
Phenotypic analysis, DEGs and highly conserved function identification. **A** Phenotypic analysis of the population. From left to right are ELR, ESS, and HU. The green, red, and purple bars represent the phenotypic values at 50, 70, and 100 weeks, respectively. **B** Phenotypic analysis of the selected samples. From left to right are ELR, ESS, and HU. The green, red, and purple bars represent the phenotypic values at 50, 70, and 100 weeks, respectively. **C** Volcano plot of DEGs. Con-up DEGs are highlighted in red and con-down DEGs are highlighted in purple. **D** Dot plot of KEGG functional enrichment in nine tissues. The left panel shows the enrichment results for con-down DEGs, and the right panel shows the enrichment results for con-up DEGs. The dot color indicates the adjusted *P* value (*P*.adjust). **E** Schematic of a eukaryotic cell nucleus and ribosome biogenesis. The nucleolus is divided into three subcompartments: the fibrillar center (FC), the dense fibrillar component (DFC), and the granular component (GC). Eukaryotic ribosome biogenesis requires RNA Polymerase I, II, and III, which transcribe ribosomal DNA (rDNA) into ribosomal RNA (rRNA), resulting in the production of 47S pre-rRNA in the nucleolus. The four rRNAs subsequently assemble with ribosomal proteins (RPs) to form the small ribosomal subunit (40S) and the large ribosomal subunit (60S). Proteins related to con-down DEGs are highlighted in dark blue. **F** The process and structure of the electron transport chain (ETC) and the TCA cycle in the mitochondria. The left panel illustrates the process of the ETC and the TCA cycle, and the right panel shows the structure of the respiratory chain complex in the inner membrane. Proteins related to con-down DEGs are highlighted in dark blue

A longitudinal tracking of the production performance in a cohort of 248 laying hens was conducted, which revealed a significant decline in ELR, ESS, and HU at 100 weeks of age compared to those at 50 and 70 weeks of age (Fig. 1A). We sampled individuals whose production performance was close to the population mean at 50, 70 and 100 weeks of age (6, 6 and 12 per group), with a specific focus on collecting more individuals at 100 weeks to mitigate the impact of high variability observed at this advanced age (Fig. 1B). From these individuals, we harvested nine tissues for RNA sequencing, including hypothalamus, pituitary, cecum, duodenum, liver, ovary, magnum, isthmus, and uterus. Quality control metrics indicated that all 216 samples achieved Q20 scores exceeding 95% and Q30 scores exceeding 90%, ensuring the reliability of downstream analyses.

To comprehensively observe the changes in different tissues across ages, we performed PCA on the samples from each tissue. The results revealed that compared to 50 and 70 weeks, all tissues exhibited more pronounced changes at 100 weeks, which suggested that the functional decline of tissues in chickens became significantly more evident between 70 and 100 weeks, leading to substantial changes in phenotypic traits (Fig. S1A).

### Ribosomal and mitochondrial dysfunction are conserved signatures of age-related production decline

To identify genes that exhibit consistent changes with phenotypic variations across tissues, we first filtered these genes to retain those whose expression levels at 70 weeks of age were intermediate between those at 50 and 100 weeks of age, ensuring a consistent expression trend across 50, 70, and 100 weeks of age. Subsequently, we further performed differential expression analysis on the gene expression between 50 and 100 weeks of age using DESeq2, to screen DEGs. We refer to these genes as consistently upregulated DEGs (con-up DEGs) and consistently downregulated DEGs (con-down DEGs), respectively. Differential expression analysis identified 842, 768, 795, 1278, 1201, 1490, 1645, 1983, and 1667 con-up DEGs in hypothalamus, pituitary, cecum, duodenum, liver, ovary, magnum, isthmus, and uterus, respectively. Additionally, the con-down DEGs amounted to 1130, 1532, 1342, 1134, 1604, 1714, 1473, 1515, and 1903 (Fig. 1C; Tables S1–S9). These results demonstrated that there were more pronounced changes in the reproductive system with age-related production decline.

To further analyze the biological functions of the con-up/down DEGs in various tissues, we conducted GO and KEGG enrichment analysis (Table S10). GO analysis revealed that the con-up DEGs across multiple tissues predominantly focused on terms related to the intrinsic component of the membrane and the cell periphery, while the con-down DEGs were primarily enriched in translation processes and ribosome-related functions (Table S10). Furthermore, it was evident that the pathways enriched by con-down DEGs in various tissues predominantly focused on ribosomal and mitochondrial activity-related pathways (Fig. 1D). Therefore, we selected genes that were significantly enriched in the ribosome pathway, oxidative phosphorylation pathway, and TCA cycle pathway across various tissues and visualized their functional localization using the KEGG Pathway database. The results revealed a significant downregulation of nearly half of the genes encoding ribosomal subunits, as well as a marked decline in the majority of functions associated with the mitochondrial respiratory chain and TCA cycle (Fig. 1E and F). Subsequently, to identify key genes representative of ribosomes and mitochondria, we performed network analysis on these genes using Cytoscape and obtained 20 hub genes (Fig. S1B and C; Table S11).

Con-up/down DEGs consistently expressed across multiple tissues were more likely to reflect universal changes associated with the aging process; therefore, we identified 87 genes that showed significant changes in all nine tissues and defined them as conserved aging-related genes (Table S12). Enrichment analysis revealed that these genes were primarily associated with ribosome function and mitochondrial function, which is consistent with the aforementioned results (Fig. S1D and E; Table S13). This suggests that impairment in ribosome and mitochondrial function may represent a potential mechanism underlying the decline in tissue function.

### Tissue-specific gene dynamics and interaction networks

**Fig. 2:**
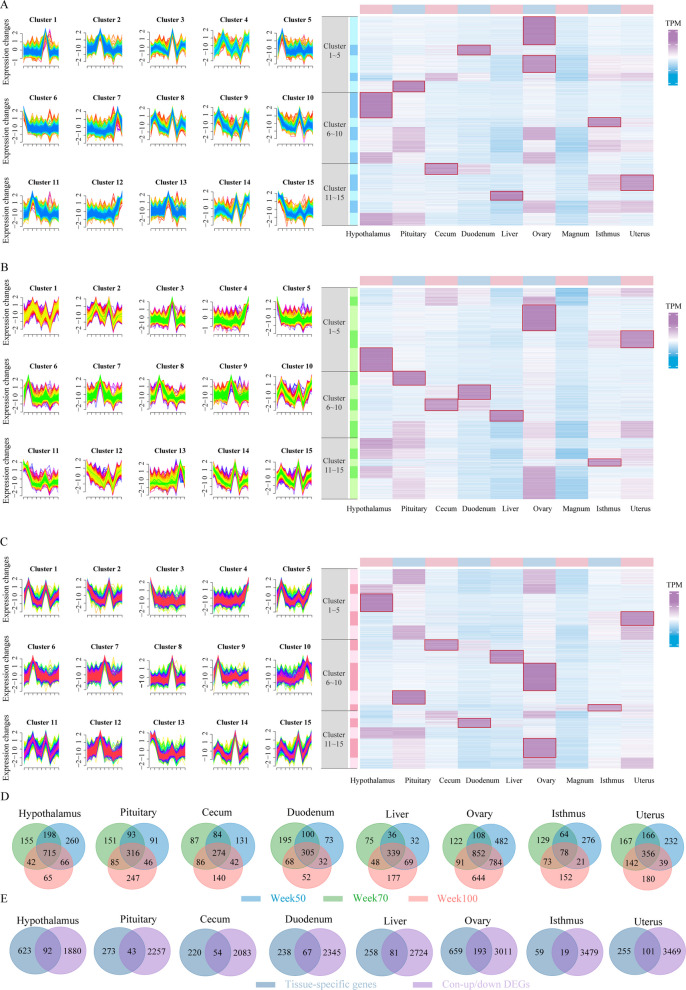
Results of the Mfuzz clustering of genes in different weeks (50, 70, 100) and Venn diagram of 9 tissues. **A–C** Mfuzz clustering and heatmap of week 50 (**A**), week 70 (**B**) and week 100 (**C**). The left panel shows the Mfuzz clustering results, while the right panel displays the corresponding expression heatmap. Genes highlighted within the red boxes are considered tissue highly expressed genes at the corresponding time point. **D** Venn diagram of tissue-specific genes of 9 tissue. Blue, green, and red represent tissue-specific genes at week 50, week 70, and week 100, respectively. The magnum is not shown because no tissue-specific genes were identified at the analyzed time points. **E** Venn diagram of tissue-specific genes in all weeks and DEGs. Dark blue and purple represent tissue-specific genes and con-up/down DEGs, respectively. The magnum is not shown because no tissue-specific genes were identified at the analyzed time points.

Decline in tissue function in chickens is a coordinated network of interrelated processes. Therefore, we hypothesized that tissue-specific gene changes during age-related production decline are interconnected and collectively reflect the underlying processes and mechanisms associated with this decline. To validate this, we performed Mfuzz clustering analysis on gene expression data from nine tissues at 50, 70, and 100 weeks of age, and visualized the differences between clusters using heatmaps. The results showed that Mfuzz clustering effectively identified tissue-specific highly expressed genes (Fig. 2A–C). We identified 715, 316, 274, 305, 339, 852, 78, and 356 tissue-specific genes that were consistently expressed across the three age points in the hypothalamus, pituitary, cecum, duodenum, liver, ovary, isthmus, and uterus, respectively (Fig. 2D). Subsequently, we intersected these genes with con-up/down DEGs in the corresponding tissues (hypothalamus, pituitary, cecum, duodenum, liver, ovary, isthmus, and uterus), resulting in 92, 43, 54, 67, 81, 193, 19, and 101 tissue-specific genes that also exhibited significant changes during age-related production decline, respectively (Fig. 2E and Table S14).

**Fig. 3:**
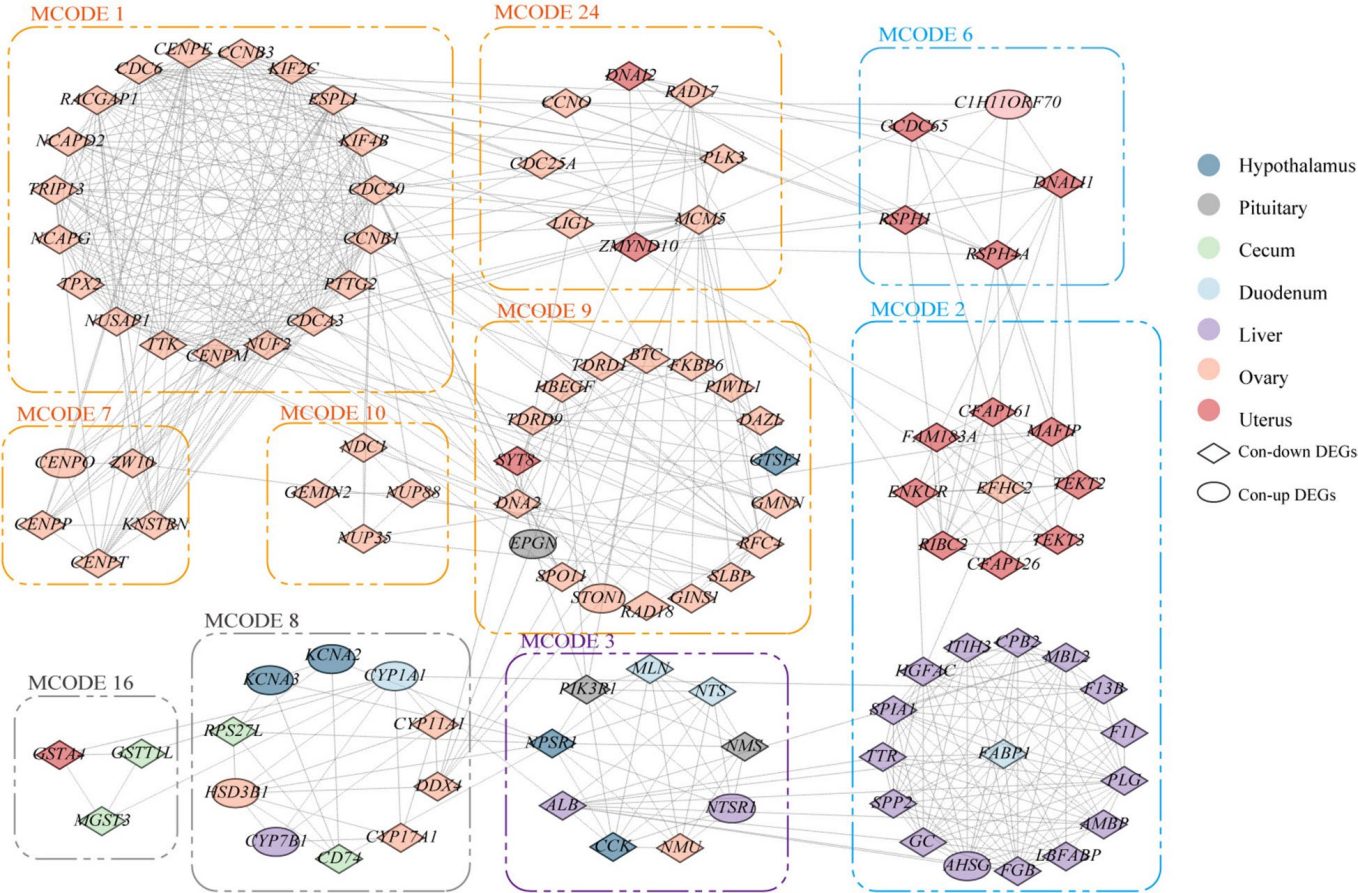
Interaction network of core modules. The deep blue circles represent tissue-specific DEGs of the hypothalamus. The gray circles represent tissue-specific DEGs of the pituitary. The green circles represent tissue-specific DEGs of the cecum. The blue circles represent tissue-specific DEGs of the duodenum. The purple circles represent tissue-specific DEGs of the liver. The orange circles represent tissue-specific DEGs of the ovary. The red circles represent tissue-specific DEGs of the uterus. The diamond represents significant downregulation of genes, the circle represents significant upregulation of genes

To elucidate the interaction networks among these genes, we used the MCODE plugin in Cytoscape to partition the genes into modules, resulting in 24 gene modules (Fig. S2). Ultimately, we identified 10 highly correlated core modules for further in-depth analysis (Fig. 3). Among these modules, modules 1, 7, 9, 10, and 24, represented by genes such as *CDC6*, *NUF2*, *ZW10*, *PIWIL1*, *GEMIN2*, and *CCNO*, play critical roles in cell division, are closely associated with ovarian function, and have been implicated in disease-related processes in multiple studies [31–35]. Meanwhile, modules 8 and 16, represented by genes such as *CYP11A1*, *GSTT1L*, and *MGST3*, are associated with the metabolism and synthesis of steroids and glutathione. Notably, *GSTT1L* gene has been found to be involved in antioxidant activity in chickens and is linked to heat tolerance and aging [36, 37]. These results demonstrate that the tissue interaction map we constructed is highly accurate and effectively reflects inter-tissue interaction effects in hens with age-related production decline, providing a foundational resource for elucidating the mechanisms underlying multi-tissue decline and identifying potential therapeutic targets.

### System-specific gene expression and pathway crosstalk during age-related production decline

**Fig. 4:**
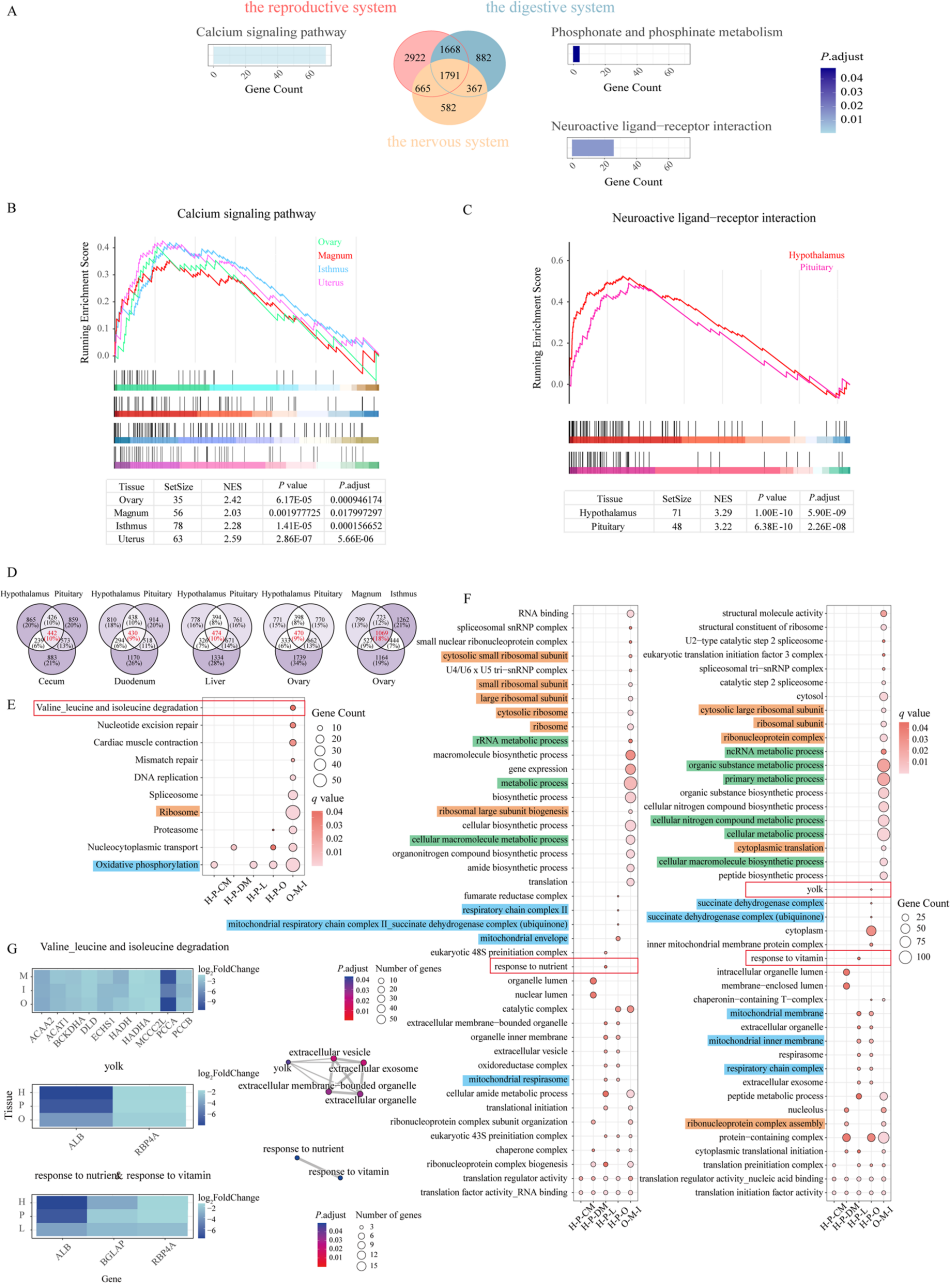
Analysis of system-specific DEGs and functional pathways. **A** Venn diagram of DEGs from different systems and the result of the KEGG analysis for system-specific DEGs. Red, blue, and yellow represent the reproductive, digestive, and nervous systems, respectively. **B** GSEA of the calcium signaling pathway in the reproductive system. Green represents the ovary, red represents the magnum, blue represents the isthmus, and purple represents the uterus. **C** GSEA of the neuroactive ligand-receptor interaction in the nervous system. Red represents hypothalamus, and pink represents pituitary. **D** Venn of H-P-CM, H-P-DM, H-P-L, H-P-O, O-M-I. **E **and** F** KEGG and GO enrichment analysis. Orange rectangles highlight pathways associated with ribosomes, blue rectangles indicate pathways related to mitochondria, and green rectangles mark pathways involved in metabolism. **G** Heatmap of specified pathway genes and pathway-associated analysis. In the left heatmap, the color indicates the log_2_-FoldChange value. In the right plot, the color represents the *P*.adjust value, and the size of the dots indicates the number of genes

Given the functional consistency of tissues within the same system, we categorized the tissues into three groups: the reproductive system (including ovary, magnum, isthmus, and uterus), digestive system (including cecum, duodenum, and liver), and nervous system (including hypothalamus and pituitary). We then identified system-specific DEGs, resulting in 2,922 reproductive system-specific DEGs, 882 digestive system-specific DEGs, and 582 nervous system-specific DEGs (Fig. 4A).

Enrichment analysis of these genes revealed significant changes in the calcium signaling pathway in the reproductive system, phosphonate and phosphinate metabolism pathway in the digestive system, and neuroactive ligand-receptor interaction pathway in the nervous system during age-related production decline in chickens (Fig. 4A). Notably, the phosphonate and phosphinate metabolism pathway in the digestive system was enriched with only four genes, *CHPT1*, *PCYT1B*, *SELENOI*, and *PCYT2*, which play crucial roles in the synthesis of phosphatidylcholine and phospholipids and are involved in key processes such as vesicle membrane formation, maintenance, and lipid metabolism, highlighting their importance in maintaining the functional stability of the digestive system.

GSEA revealed significant upregulation of calcium signaling in the reproductive system (Fig. 4B). Because calcium and phosphorus are crucial elements for eggshell formation [38], their alterations may contribute to the increased heterogeneity of egg quality during the late laying period. In the nervous system, we observed an upregulation in the neuroactive ligand-receptor interaction pathway (Fig. 4C). Gene functional annotation demonstrated that genes associated with the GH and HPA axes exhibited upward trends, collectively reinforcing the age-related production decline process [39, 40] and may contribute to reduced feed intake through HPA axis upregulation [41].

To further investigate inter-tissue interaction changes, we reclassified the nine tissues into four functional axes: 1) Brain-gut axis: hypothalamus-pituitary-cecum (H-P-CM) and hypothalamus-pituitary-duodenum (H-P-DM); 2) Brain-liver axis: hypothalamus-pituitary-liver (H-P-L); 3) Gonadal axis: hypothalamus-pituitary-ovary (H-P-O); and 4) Eggwhite-eggshell formation axis: ovary-magnum-isthmus (O-M-I). We identified 442, 430, 474, 470, and 1069 con-up/down DEGs in these respective groups (Fig. 4D). Both KEGG and GO enrichment analyses revealed substantial gene enrichment in ribosomal, mitochondrial, and various metabolic pathways, which is consistent with our previous findings (Fig. 4E and F). Notably, specific enrichment results included valine, leucine and isoleucine degradation in O-M-I, yolk in H-P-O, and responses to nutrients and vitamins in H-P-L (Fig. 4E and F). Moreover, the H-P-O axis, a critical neuroendocrine regulator closely associated with laying performance in hens, plays a pivotal role in controlling both egg production efficiency and egg quality, thus garnering significant research attention. Notably, the *ALB* gene identified in the yolk formation pathway has been extensively validated across multiple studies as being functionally linked to egg quality traits [42, 43]. The analysis of pathway-associated genes demonstrated consistent downward trends, indicating reduced nutrient absorption, decreased amino acid degradation capacity, and diminished yolk production capability in late-laying hens (Fig. 4G), which may represent key factors underlying the decline in egg production rate and egg quality during the late laying period.

### Identification of key genes associated with age-related production decline

To identify a gene set representative of the age-related production decline process in chickens, we integrated three groups of genes: 87 con-up/down DEGs expressed across all nine tissues, 20 hub genes playing core roles in ribosome and mitochondrial function, and 106 con-up/down DEGs from tissue-specific core modules, resulting in a total of 207 candidate genes. To ensure that these genes were associated with key production traits in chickens, we analyzed gene expression data from another cohort consisting of 248 100-week-old laying hens across seven tissues (hypothalamus, pituitary, cecum, duodenum, liver, ovary, uterus). Through this analysis, we identified 152 genes that were significantly correlated with key phenotypes (ELR, ESS, or HU) (*P* < 0.05) (Table S15).

Subsequently, we further filtered these genes using gene expression data from nine tissues of 100-week-old laying hens. The samples were divided into forced- and non-forced-molting groups. Given that forced molting enhances production performance and exhibits anti-aging trends in chickens, we identified 51 genes showing significant expression changes in at least four tissues (*P* < 0.05) and defined them as key genes associated with age-related production decline in chickens (Table S16).

**Fig. 5:**
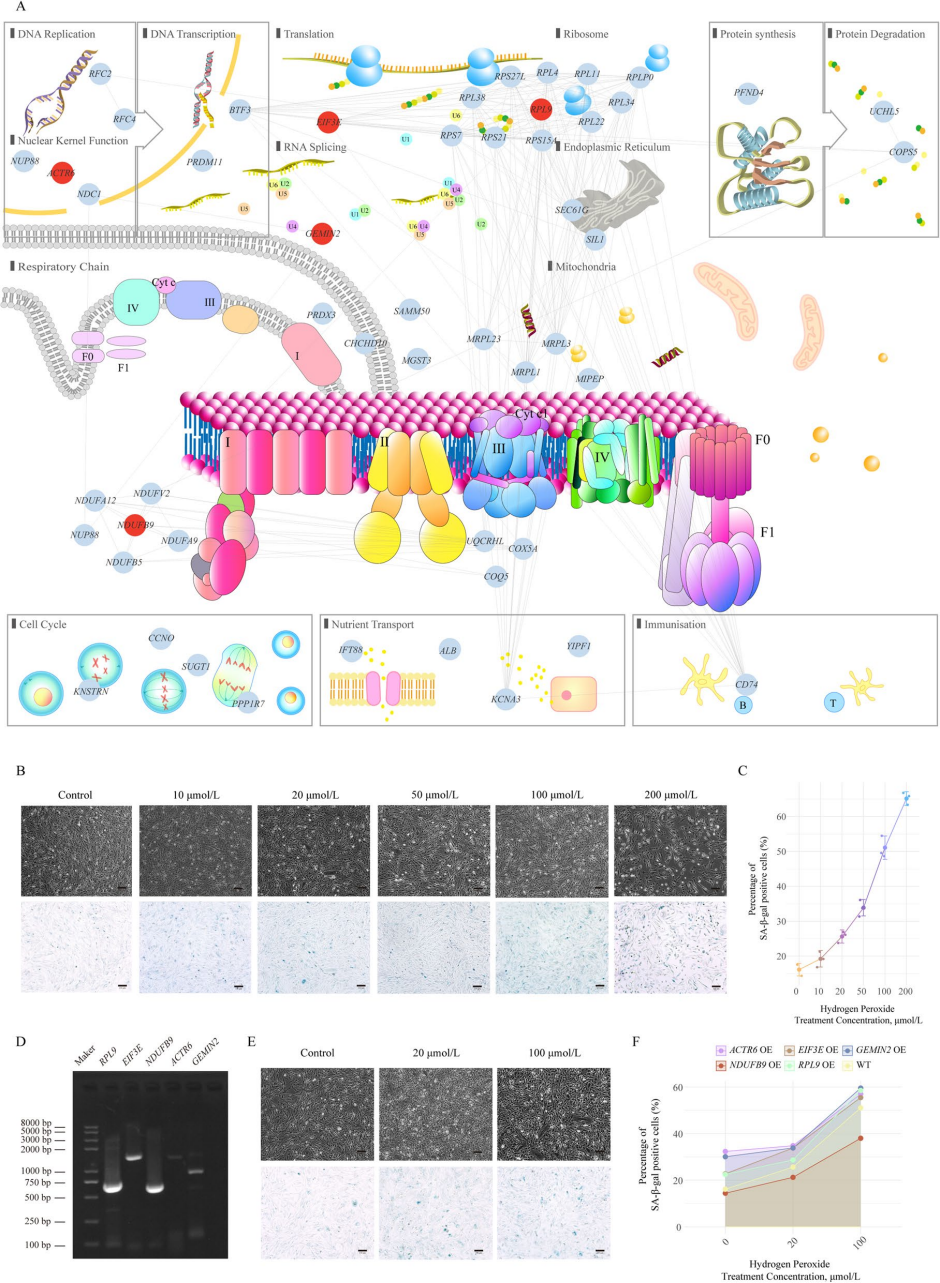
Results of hydrogen peroxide treatment on positive cells and the gene network of 51 key aging-related genes. **A** Gene network and functional distribution map of the 51 key aging-related genes. Genes highlighted in red represent the overexpressed genes: *RPL9*, *ACTR6*, *NDUFB9*, *EIF3E*, and *GEMIN2*. The connections between genes were generated using network analysis in Cytoscape. **B** β-Galactosidase staining result image of wild-type DF-1 cells. From left to right: control group (no hydrogen peroxide treatment) and hydrogen peroxide-treated groups at 10, 20, 50, 100, and 200 μmol/L concentrations. **C** Line graph of the percentage of SA-β-Gal positive cells in wild-type DF-1 cells. **D** PCR gel image of the target fragment. **E** β-galactosidase staining result image of *NDUFB9*-overexpressing DF-1 cells. **F** Line graph of the percentage of SA-β-Gal positive cells in *RPL9*-, *ACTR6*-, *NDUFB9*-, *EIF3E*-, *GEMIN2*-overexpressing DF-1 cells with wild-type DF-1 cells. Pink, brown, blue, red, green, and yellow represent *ACTR6*-, *EIF3E*-, *GEMIN2*-, *NDUFB9*-, *RPL9*-overexpressing cells, and WT cells, respectively

We aimed to identify key genes capable of delaying aging from the 51 candidate genes screened using the aforementioned approach. Functional annotation of these genes revealed that most were involved in DNA replication, transcription, translation, RNA splicing, protein synthesis and degradation, endoplasmic reticulum and mitochondrial structure, cell cycle, and molecular transport (Fig. 5A). To identify potential targets for delaying aging, we focused on genes related to gene expression processes and mitochondrial energy production. Specifically, we selected the following genes for further validation: *ACTR6*, which maintains nucleolar function within the nucleolus; *EIF3E*, which is responsible for transcription initiation during the transcription process; *RPL9*, which plays a role in maintaining ribosomal structure; *GEMIN2*, which is involved in mRNA splicing; and *NDUFB9*, which is associated with the function of the mitochondrial respiratory chain complex I.

### *NDUFB9*-mediated mitochondrial complex I enhancement mitigates oxidative stress-driven senescence

First, we treated DF-1 cells with hydrogen peroxide, a well-known free radical that induces cellular senescence and is widely used to establish cellular senescence models [44, 45], at different concentrations (10, 20, 50, 100, and 200 μmol/L) for 24 h. Subsequently, we used a β-galactosidase staining kit to detect the proportion of senescent cells, which revealed that the proportion of β-Gal-positive cells increased gradually from 16% in the control group to 66% in the 200 μmol/L hydrogen peroxide-treated group, accompanied by significant changes in cell morphology (Fig. 5B and C). Additionally, using a Mitochondrial Membrane Potential and Apoptosis Detection staining kit, we observed that the number of cells exhibiting green fluorescence increased with higher hydrogen peroxide concentrations, whereas the number of cells exhibiting red fluorescence decreased (Fig. S3B), further confirming the successful induction of a cellular senescence model by hydrogen peroxide.

Since the five selected genes exhibited a consistent downward trend across the nine tissues over time, we chose to overexpress these genes to explore their potential as targets for delaying aging (Fig. S3A). Using the designed primers (Table S17), we successfully amplified the target fragments of the selected genes and constructed the overexpression plasmids (Fig. 5D). After transfecting the genes into cells, we obtained DF-1 cells overexpressing the target genes. Subsequently, we used β-galactosidase staining to detect changes in the proportion of senescent cells under untreated, 20 μmol/L hydrogen peroxide-treated, and 100 μmol/L hydrogen peroxide-treated conditions. The results showed that the overexpression of *ACTR6*, *EIF3E*, *RPL9*, or *GEMIN2* individually led to a significant increase in the number of senescent cells. After treatment with 20 μmol/L or 100 μmol/L hydrogen peroxide, the proportion of senescent cells remained elevated (Fig. 5F, Fig. S3C). In striking contrast, overexpression of *NDUFB9*, a gene linked to mitochondrial respiratory chain function, markedly reduced senescence. Following 24-h hydrogen peroxide exposure across varying concentrations, *NDUFB9*-overexpressing cells maintained significantly fewer senescent cells than wild-type DF-1 controls. Notably, at 100 μmol/L hydrogen peroxide, senescent cells dropped below 50% (Fig. 5E and F), suggesting that modulating mitochondrial respiratory activity could be a viable strategy to mitigate cellular senescence.

Integrating the above experimental results, we conclude that impaired mitochondrial energy production is a critical factor contributing to cellular dysfunction, whereas enhanced mitochondrial function, particularly in the respiratory chain, plays a significant role in delaying cellular senescence and tissue functional decline. Therefore, genes associated with the mitochondrial respiratory chain complex I may serve as key targets for delaying aging.

## Discussion

In recent years, the rapid development of large-scale data collection and analysis technology has significantly advanced research in the field of aging. Although research on aging in chickens has increased, comprehensive studies analyzing tissue-specific changes and interactions associated with tissue function decline are still lacking. Moreover, the specific gene sets related to hens with age-related production decline and key targets for aging interventions remain unidentified. To address these gaps, this study explored the spatiotemporal specificity of tissue function decline in chickens through a multidimensional analysis of gene expression profiles across multiple age points and tissues.

Our analysis of DEGs across nine tissues revealed that the reproductive system underwent the most pronounced functional changes during the late laying period, suggesting that tissue function decline may have originated in this system. This finding aligns with those reported by Liu et al. [46]. Similar patterns have been observed in other species, with studies showing that the female reproductive system in mammals undergoes physiological aging more rapidly than the other systems [47]. In humans, reproductive health significantly declines after the age of 35, accompanied by a notable reduction in pregnancy rates [48].

Furthermore, in the system-specific analysis of DEGs, we found that the digestive and reproductive systems exhibited system-specific DEGs enriched in pathways related to phosphonate and phosphinate metabolism and calcium signaling, respectively. Calcium and phosphorus are critical elements for eggshell formation and are indispensable for various processes associated with eggshell development [49, 50]. Deficiencies in these elements can lead to severe consequences including reduced appetite, osteoporosis, and weight loss in aging poultry. During the late laying period, eggshell quality significantly declines, and the rate of broken eggs increases [7, 8, 10]. These findings suggested that dysregulation in phosphorus and calcium metabolism not only contributed to the increased variability in egg-laying traits but also served as a critical indicator of declining tissue function, particularly in the reproductive and digestive systems.

In the analysis of the H-P-O axis and O-M-I axis, which are closely associated with egg production, we observed that most pathways were significantly related to ribosomal and mitochondrial functions. This implies that these biological processes may play a pivotal role in the decline in tissue function. Through the analysis of the gene interaction network and expression profile in the neuroactive ligand-receptor interaction pathway, we observed a significant increase in the expression of genes associated with the GH and HPA axes. Numerous studies have confirmed that GH levels increase in aging individuals and elevated GH levels are known to promote aging. Furthermore, upregulation of the HPA axis is associated with decreased feed intake. While some studies suggest that the secretion of sex hormones decreases as aging progresses [51–53], others indicate no significant changes in sex hormone secretion [54, 55]. The changes in sex hormones associated with aging exhibit specific temporal and species-related characteristics, which is consistent with our observations based on multi-tissue sampling across different time points.

Because the pathways enriched by DEGs in the reproductive system and other tissues were predominantly associated with ribosomes and mitochondria, we further analyzed the genes functioning in these organelles. Our results indicated a widespread decline in ribosomal and mitochondrial function across multiple tissues, consistent with previous studies. Studies have confirmed that the ribosome function decreases in aging cells [56, 57], which subsequently leads to cell cycle arrest and promotes the aging process [58]. Additionally, studies suggest that the mitochondrial function of aging cells may be impaired, resulting in decreased efficiency of ATP production and an increase in the AMP/ATP ratio [59, 60]. Mitochondrial dysfunction can also contribute to the accumulation of reactive oxygen species (ROS) [61, 62], decreased antioxidant capacity [63–67], and calcium ion imbalance [68].

Meanwhile, the proteasome and spliceosome also appeared in numerous results, showing a trend of functional decline. Proteasomes and spliceosomes play crucial roles in regulating the cell cycle [69–71], apoptosis [70, 72, 73], protein quality control [74–76], among other functions. In contrast to our results, some studies suggest that the primary spliceosome complex proteins significantly increase with age [77]. This discrepancy may be attributed to differences in the developmental stages, as the compensatory effect of the body disrupts the activity of spliceosomes.

The four aspects of functional impairment mentioned above are closely linked to DNA expression, macromolecular synthesis metabolism, etc. Therefore, we speculated that this may be one of the main reasons for the decline in tissue function. Continuous DNA expression damage can induce aging, leading to the senescence-associated secretory phenotype (SASP) and creating a vicious cycle [78, 79]. This may represent a key entry point for delaying age-related production decline in chickens. Therefore, we selected five genes, *ACTR6*, *GEMIN2*, *RPL9*, *EIF3E*, and *NDUFB9*, which play pivotal roles in key pathways for validation in a DF-1 cell senescence model induced by hydrogen peroxide.

Our findings demonstrated that overexpression of *NDUFB9*, a key gene in the mitochondrial respiratory chain, significantly delays aging. This highlighted the potential of targeting mitochondrial function as a single regulatory mechanism to mitigate aging, with broader implications for understanding age-related declines in cellular and tissue function. At the same time, studies have also suggested that *NDUFB9* gene may play a role in cell death, synaptic plasticity, and cell adhesion [80]. *NDUFB9* is a subunit of inner mitochondrial complex I. Malfunctions in complex I are the primary cause of oxidative phosphorylation disorders and are linked to various illnesses [81]. Furthermore, mutations in *NDUFB9* can lead to complex I deficiency, which has been reported to promote tumor metastasis [82–84]. This implies that the mitochondrial respiratory chain may play a crucial role in slowing down aging as a potential target for intervening or revealing aging in chickens. Overall, this study provides a detailed analysis of the aging-induced productivity decline in chickens, revealing significant tissue-specific changes across 9 tissues at three distinct age points. By compiling the first aging gene set for chickens, we have identified several key genes and potential targets for intervening in age-related production decline. These findings not only contribute to a deeper understanding of the molecular mechanisms underlying age-related production decline in poultry but also pave the way for future research into therapeutic strategies aimed at mitigating age-related declines in reproductive and metabolic functions.

## Conclusion

We constructed gene expression profiles across multiple chicken tissues and identified that the reproductive system undergoes the most significant changes with age. Additionally, we observed a decline in the functional activity of ribosome and mitochondria across multiple tissues. Through a comprehensive analysis of gene expression and phenotypic data, we identified 51 aging-related genes in chickens. Finally, functional validation in a DF-1 cellular senescence model revealed that *NDUFB9*, a gene associated with the mitochondrial respiratory chain, may serve as a potential target for delaying aging, providing further evidence for the critical role of mitochondrial function in organismal aging. Our study advances the understanding of the genetic regulatory mechanisms underlying tissue function decline in chickens and provides a solid theoretical foundation for molecular breeding, while helping to fill the gap in defined gene sets associated with age-related production decline in poultry.

## Supplementary Information

Additional file 1: Table S1 DESeq2 results for the hypothalamus (50 vs. 100 weeks). Table S2 DESeq2 results for the pituitary (50 vs. 100 weeks). Table S3 DESeq2 results for the cecum (50 vs. 100 weeks). Table S4 DESeq2 results for the duodenum (50 vs. 100 weeks). Table S5 DESeq2 results for the liver (50 vs. 100 weeks). Table S6 DESeq2 results for the ovary (50 vs. 100 weeks). Table S7 DESeq2 results for the magnum (50 vs. 100 weeks). Table S8 DESeq2 results for the isthmus (50 vs. 100 weeks). Table S9 DESeq2 results for the uterus (50 vs. 100 weeks). Table S10 GO and KEGG results of DEGs in 9 tissues. Table S11 Hub genes of ribosome and mitochondria. Table S12 87 DEGs in 9 tissues. Table S13 GO and KEGG results of 87 DEGs. Table S14 tissue-specific DEGs. Table S15 Correlation results between gene set and ELR, ESS, HU. Table S16 Aging gene set. Table S17 The primer information of *RPL9*, *EIF3E*, *NDUFB9*, *ACTR6*, *GEMIN2*.

Additional file 2: Fig. S1. Integrated analysis of age-dependent gene expression and functional networks. Fig. S2. The result of MCODE analysis of the tissue-specific DEGs. Fig. S3. Line graph and results of cell staining for mitochondrial membrane potential, apoptosis, and SA-β-Gal staining.

## Data Availability

In this study, the generated transcriptome data have been deposited in the NCBI BioProject database (https://www.ncbi.nlm.nih.gov/bioproject/) under the accession number PRJNA1240437. The dataset comprises transcriptome profiles from Rhode Island Red laying hens at 50, 70, and 100 weeks of age, covering nine tissues (hypothalamus, pituitary, cecum, duodenum, liver, ovary, magnum, isthmus, and uterus). Data associated with forced molting are not included.

## Abbreviations

con-down DEGs: Consistently downregulated differentially expressed genes
con-up DEGs: Consistently upregulated differentially expressed genes
DEGs: Differentially expressed genes
ELR: Egg-laying rate
ESS: Eggshell strength
FDR: False discovery rate
GO: Gene Ontology
GSEA: Gene set enrichment analysis
HU: Haugh unit
H-P-CM: Hypothalamus-pituitary-cecum
H-P-DM: Hypothalamus-pituitary-duodenum
H-P-L: Hypothalamus-pituitary-liver
H-P-O: Hypothalamus-pituitary-ovary
KEGG: Kyoto Encyclopedia of Genes and Genomes
O-M-I: Ovary-magnum-isthmus
PCA: Principal component analysis
SASP: Senescence-associated secretory phenotype

## References

[1] López-Otín C, Blasco MA, Partridge L, Serrano M, Kroemer G. Hallmarks of aging: an expanding universe. Cell. 2023;186:243–78. 10.1016/j.cell.2022.11.001. 36599349 10.1016/j.cell.2022.11.001

[2] Roy AL, Sierra F, Howcroft K, Singer DS, Sharpless N, Hodes RJ, et al. A blueprint for characterizing senescence. Cell. 2020;183:1143–6. 10.1016/j.cell.2020.10.032. 33128870 10.1016/j.cell.2020.10.032 PMC8364378

[3] Avelar RA, Ortega JG, Tacutu R, Tyler EJ, Bennett D, Binetti P, et al. A multidimensional systems biology analysis of cellular senescence in aging and disease. Genome Biol. 2020;21:91. 10.1186/s13059-020-01990-9. 32264951 10.1186/s13059-020-01990-9 PMC7333371

[4] Saul D, Kosinsky RL, Atkinson EJ, Doolittle ML, Zhang X, LeBrasseur NK, et al. A new gene set identifies senescent cells and predicts senescence-associated pathways across tissues. Nat Commun. 2022;13:4827. 10.1038/s41467-022-32552-1. 35974106 10.1038/s41467-022-32552-1 PMC9381717

[5] Yuan J, Chang SY, Yin SG, Liu ZY, Cheng X, Liu XJ, et al. Two conserved epigenetic regulators prevent healthy ageing. Nature. 2020;579:118–22. 10.1038/s41586-020-2037-y. 32103178 10.1038/s41586-020-2037-y

[6] Bain MM, Nys Y, Dunn IC. Increasing persistency in lay and stabilising egg quality in longer laying cycles. What are the challenges? Br Poult Sci. 2016;57:330–8. 10.1080/00071668.2016.1161727. 26982003 10.1080/00071668.2016.1161727 PMC4940894

[7] Al-batshan HA, Scheideler SE, Black BL, Garlich JD, Anderson KE. Duodenal calcium uptake, femur ash, and eggshell quality decline with age and increase following Molt1. Poult Sci. 1994;73:1590–6. 10.3382/ps.0731590. 7816734 10.3382/ps.0731590

[8] Joyner CJ, Peddie MJ, Taylor TG. The effect of age on egg production in the domestic hen. Gen Comp Endocrinol. 1987;65:331–6. 10.1016/0016-6480(87)90117-1. 3557097 10.1016/0016-6480(87)90117-1

[9] Molnár A, Maertens L, Ampe B, Buyse J, Kempen I, Zoons J, et al. Changes in egg quality traits during the last phase of production: is there potential for an extended laying cycle? Br Poult Sci. 2016;57:842–7. 10.1080/00071668.2016.1209738. 27385085 10.1080/00071668.2016.1209738

[10] Travel A, Nys Y, Bain M. Effect of hen age, moult, laying environment and egg storage on egg quality. In: Nys Y, Bain M, Van Immerseel F, editors. Improving the safety and quality of eggs and egg products. Woodhead Publishing; 2011. p. 300–29. 10.1533/9780857093912.3.300. Cited 2024 Oct 5.

[11] Li G, Feng Y, Cui J, Hou Q, Li T, Jia M, et al. The ionome and proteome landscape of aging in laying hens and relation to egg white quality. Sci China Life Sci. 2023;66:2020–40. 10.1007/s11427-023-2413-4. 37526911 10.1007/s11427-023-2413-4

[12] Solomon S. The oviduct in chaos. Worlds Poult Sci J. 2002;58:41–8. 10.1079/WPS20020006.

[13] Solomon S, Solomon S. Egg and eggshell quality. 1997. https://www.semanticscholar.org/paper/Egg-and-Eggshell-Quality-Solomon-Solomon/8abb84307591b7e3066fc3318863d4297f556320. Accessed 2 Sept 2024.

[14] Chang X, Wang B, Zhang H, Qiu K, Wu S. The change of albumen quality during the laying cycle and its potential physiological and molecular basis of laying hens. Poult Sci. 2024;103:104004. 10.1016/j.psj.2024.104004. 39067125 10.1016/j.psj.2024.104004 PMC11331942

[15] Martin M. Cutadapt removes adapter sequences from high-throughput sequencing reads. EMBnet J. 2011;17:10–2. 10.14806/ej.17.1.200.

[16] Thompson O, von Meyenn F, Hewitt Z, Alexander J, Wood A, Weightman R, et al. Low rates of mutation in clinical grade human pluripotent stem cells under different culture conditions. Nat Commun. 2020;11:1528. 10.1038/s41467-020-15271-3. 32251294 10.1038/s41467-020-15271-3 PMC7089967

[17] Kovaka S, Zimin AV, Pertea GM, Razaghi R, Salzberg SL, Pertea M. Transcriptome assembly from long-read RNA-seq alignments with StringTie2. Genome Biol. 2019;20:278. 10.1186/s13059-019-1910-1. 31842956 10.1186/s13059-019-1910-1 PMC6912988

[18] Pertea M, Kim D, Pertea GM, Leek JT, Salzberg SL. Transcript-level expression analysis of RNA-seq experiments with HISAT, StringTie and Ballgown. Nat Protoc. 2016;11:1650–67. 10.1038/nprot.2016.095. 27560171 10.1038/nprot.2016.095 PMC5032908

[19] Pertea M, Pertea GM, Antonescu CM, Chang TC, Mendell JT, Salzberg SL. StringTie enables improved reconstruction of a transcriptome from RNA-seq reads. Nat Biotechnol. 2015;33:290–5. 10.1038/nbt.3122. 25690850 10.1038/nbt.3122 PMC4643835

[20] Love MI, Huber W, Anders S. Moderated estimation of fold change and dispersion for RNA-seq data with DESeq2. Genome Biol. 2014;15:550. 10.1186/s13059-014-0550-8. 25516281 10.1186/s13059-014-0550-8 PMC4302049

[21] Benjamini Y, Hochberg Y. Controlling the false discovery rate: a practical and powerful approach to multiple testing. J R Stat Soc Series B Stat Methodol. 1995;57:289–300. 10.1111/j.2517-6161.1995.tb02031.x.

[22] Binns D, Dimmer E, Huntley R, Barrell D, O’Donovan C, Apweiler R. QuickGO: a web-based tool for Gene Ontology searching. Bioinformatics. 2009;25:3045–6. 10.1093/bioinformatics/btp536. 19744993 10.1093/bioinformatics/btp536 PMC2773257

[23] Kanehisa M, Araki M, Goto S, Hattori M, Hirakawa M, Itoh M, et al. KEGG for linking genomes to life and the environment. Nucleic Acids Res. 2008;36:D480-484. 10.1093/nar/gkm882. 18077471 10.1093/nar/gkm882 PMC2238879

[24] Wu T, Hu E, Xu S, Chen M, Guo P, Dai Z, et al. ClusterProfiler 4.0: a universal enrichment tool for interpreting omics data. Innovation. 2021;2:100141. 10.1016/j.xinn.2021.100141. 34557778 10.1016/j.xinn.2021.100141 PMC8454663

[25] Yu G, Wang LG, Han Y, He QY. ClusterProfiler: an R package for comparing biological themes among gene clusters. OMICS. 2012;16:284–7. 10.1089/omi.2011.0118. 22455463 10.1089/omi.2011.0118 PMC3339379

[26] Subramanian A, Tamayo P, Mootha VK, Mukherjee S, Ebert BL, Gillette MA, et al. Gene set enrichment analysis: a knowledge-based approach for interpreting genome-wide expression profiles. Proc Natl Acad Sci U S A. 2005;102:15545–50. 10.1073/pnas.0506580102. 16199517 10.1073/pnas.0506580102 PMC1239896

[27] Kumar L, Futschik ME. Mfuzz: a software package for soft clustering of microarray data. Bioinformation. 2007;2:5–7. 10.6026/97320630002005 10.6026/97320630002005 PMC2139991 18084642

[28] Snel B, Lehmann G, Bork P, Huynen MA. STRING: a web-server to retrieve and display the repeatedly occurring neighbourhood of a gene. Nucleic Acids Res. 2000.15;28(18):3442-4. 10.1093/nar/28.18.3442 10.1093/nar/28.18.3442 PMC110752 10982861

[29] Saito R, Smoot ME, Ono K, Ruscheinski J, Wang PL, Lotia S, et al. A travel guide to Cytoscape plugins. Nat Methods. 2012;9:1069–76. 10.1038/nmeth.2212. 23132118 10.1038/nmeth.2212 PMC3649846

[30] Chin CH, Chen SH, Wu HH, Ho CW, Ko MT, Lin CY. cytoHubba: identifying hub objects and sub-networks from complex interactome. BMC Syst Biol. 2014;8:S11. 10.1186/1752-0509-8-S4-S11. 25521941 10.1186/1752-0509-8-S4-S11 PMC4290687

[31] Abdelhamid RF, Ogawa K, Beck G, Ikenaka K, Takeuchi E, Yasumizu Y, et al. piRNA/PIWI protein complex as a potential biomarker in Sporadic Amyotrophic Lateral Sclerosis. Mol Neurobiol. 2022;59:1693–705. 10.1007/s12035-021-02686-2. 35015250 10.1007/s12035-021-02686-2 PMC8882100

[32] Ishikawa Y, Fukue H, Iwakami R, Ikeda M, Iemura K, Tanaka K. Fibrous corona is reduced in cancer cell lines that attenuate microtubule nucleation from kinetochores. Cancer Sci. 2025;116:420–31. 10.1111/cas.16406. 39604214 10.1111/cas.16406 PMC11786318

[33] Lim N, Townsend PA. Cdc6 as a novel target in cancer: oncogenic potential, senescence and subcellular localisation. Int J Cancer. 2020;147:1528–34. 10.1002/ijc.32900. 32010971 10.1002/ijc.32900 PMC7496346

[34] Núnez-Ollé M, Jung C, Terré B, Balsiger NA, Plata C, Roset R, et al. Constitutive cyclin O deficiency results in penetrant hydrocephalus, impaired growth and infertility. Oncotarget. 2017;8:99261–73. 10.18632/oncotarget.21818. 29245899 10.18632/oncotarget.21818 PMC5725090

[35] Yamamoto S, Takayama K-I, Obinata D, Fujiwara K, Ashikari D, Takahashi S, et al. Identification of new octamer transcription factor 1-target genes upregulated in castration-resistant prostate cancer. Cancer Sci. 2019;110:3476–85. 10.1111/cas.14183. 31454442 10.1111/cas.14183 PMC6825001

[36] Liu X, Ma Z, Wang Y, Jia H, Wang Z, Zhang L. Heat stress exposure cause alterations in intestinal microbiota, transcriptome, and metabolome of broilers. Front Microbiol. 2023;14:1244004. 10.3389/fmicb.2023.1244004. 37795292 10.3389/fmicb.2023.1244004 PMC10547010

[37] Zhao CX, Liu JN, Li BQ, Ren D, Chen X, Yu J, et al. Multiscale construction of bifunctional electrocatalysts for long-Lifespan rechargeable Zinc–Air batteries. Adv Funct Mater. 2020;30:2003619. 10.1002/adfm.202003619.

[38] Klein L. Direct measurement of bone resorption and calcium conservation during vitamin D deficiency or hypervitaminosis D. Proc Natl Acad Sci U S A. 1980;77:1818–22. 10.1073/pnas.77.4.1818. 6246506 10.1073/pnas.77.4.1818 PMC348599

[39] Aguiar-Oliveira MH, Bartke A. Growth hormone deficiency: health and longevity. Endocr Rev. 2019;40:575–601. 10.1210/er.2018-00216. 30576428 10.1210/er.2018-00216 PMC6416709

[40] Xing Y, Xuan F, Wang K, Zhang H. Aging under endocrine hormone regulation. Front Endocrinol (Lausanne). 2023;14:1223529. 10.3389/fendo.2023.1223529. 37600699 10.3389/fendo.2023.1223529 PMC10433899

[41] Paeger L, Karakasilioti I, Altmüller J, Frommolt P, Brüning J, Kloppenburg P. Antagonistic modulation of NPY/AgRP and POMC neurons in the arcuate nucleus by noradrenalin. Elife. 2017;6:e25770. 10.7554/eLife.25770. 28632132 10.7554/eLife.25770 PMC5478265

[42] Eltahan HM, Cho S, Rana MM, Saleh AA, Elkomy AE, Wadaan MAM, et al. Dietary exogenous phytase improve egg quality, reproductive hormones, and prolongs the lifetime of the aging Hy-Line brown laying hens fed nonphytate phosphorus. Poult Sci. 2023;102:102895. 10.1016/j.psj.2023.102895. 37441904 10.1016/j.psj.2023.102895 PMC10362347

[43] Zhou W, Miao S, Zhu M, Dong X, Zou X. Effect of glycine nano-selenium supplementation on production performance, egg quality, serum biochemistry, oxidative status, and the intestinal morphology and absorption of laying hens. Biol Trace Elem Res. 2021;199:4273–83. 10.1007/s12011-020-02532-x. 33615395 10.1007/s12011-020-02532-x

[44] Oh S, Rhee D-Y, Batsukh S, Son KH, Byun K. High-intensity focused ultrasound increases collagen and elastin fiber synthesis by modulating caveolin-1 in aging skin. Cells. 2023;12:2275. 10.3390/cells12182275. 37759497 10.3390/cells12182275 PMC10527789

[45] Yoon DS, Lee KM, Choi Y, Ko EA, Lee NH, Cho S, et al. TLR4 downregulation by the RNA-binding protein PUM1 alleviates cellular aging and osteoarthritis. Cell Death Differ. 2022;29:1364–78. 10.1038/s41418-021-00925-6. 35034101 10.1038/s41418-021-00925-6 PMC9287402

[46] Liu X, Lin X, Mi Y, Li J, Zhang C. Grape seed proanthocyanidin extract prevents ovarian aging by inhibiting oxidative stress in the hens. Oxid Med Cell Longev. 2018;2018:9390810. 10.1155/2018/9390810. 29541349 10.1155/2018/9390810 PMC5818927

[47] Stoop D, Cobo A, Silber S. Fertility preservation for age-related fertility decline. Lancet. 2014;384:1311–9. 10.1016/S0140-6736(14)61261-7. 25283572 10.1016/S0140-6736(14)61261-7

[48] Silber SJ, Kato K, Aoyama N, Yabuuchi A, Skaletsky H, Fan Y, et al. Intrinsic fertility of human oocytes. Fertil Steril. 2017;107:1232–7. 10.1016/j.fertnstert.2017.03.014. 28433372 10.1016/j.fertnstert.2017.03.014

[49] An SH, Kim DW, An BK. Effects of dietary calcium levels on productive performance, eggshell quality and overall calcium status in aged laying hens. Asian-Australas J Anim Sci. 2016;29:1477–82. 10.5713/ajas.15.0655. 26954217 10.5713/ajas.15.0655 PMC5003974

[50] Rama Rao SV, Raju MVLN, Reddy MR, Pavani P. Interaction between dietary calcium and non-phytate phosphorus levels on growth, bone mineralization and mineral excretion in commercial broilers. Anim Feed Sci Technol. 2006;131:135–50. 10.1016/j.anifeedsci.2006.02.011.

[51] Bronson FH, Desjardins C. Reproductive failure in aged CBF1 male mice: interrelationships between pituitary gonadotropic hormones, testicular function, and mating success. Endocrinology. 1977;101:939–45. 10.1210/endo-101-3-939. 891473 10.1210/endo-101-3-939

[52] Greenspan SL, Klibanski A, Rowe JW, Elahi D. Age alters pulsatile prolactin release: influence of dopaminergic inhibition. Am J Physiol. 1990;258:E799-804. 10.1152/ajpendo.1990.258.5.E799. 2333989 10.1152/ajpendo.1990.258.5.E799

[53] Iranmanesh A, Mulligan T, Veldhuis JD. Mechanisms subserving the physiological nocturnal relative hypoprolactinemia of healthy older men: dual decline in prolactin secretory burst mass and basal release with preservation of pulse duration, frequency, and interpulse interval–a general clinical research center study. J Clin Endocrinol Metab. 1999;84:1083–90. 10.1210/jcem.84.3.5514. 10084599 10.1210/jcem.84.3.5514

[54] Elmlinger MW, Dengler T, Weinstock C, Kuehnel W. Endocrine alterations in the aging male. Clin Chem Lab Med. 2003;41:934–41. 10.1515/CCLM.2003.142. 12940521 10.1515/CCLM.2003.142

[55] Jacques C, Schlienger JL, Kissel C, Kuntzmann F, Sapin R. TRH-induced TSH and prolactin responses in the elderly. Age Ageing. 1987;16:181–8. 10.1093/ageing/16.3.181. 3111192 10.1093/ageing/16.3.181

[56] Nishimura K, Kumazawa T, Kuroda T, Katagiri N, Tsuchiya M, Goto N, et al. Perturbation of ribosome biogenesis drives cells into senescence through 5S RNP-mediated p53 activation. Cell Rep. 2015;10:1310–23. 10.1016/j.celrep.2015.01.055. 25732822 10.1016/j.celrep.2015.01.055

[57] Tiku V, Jain C, Raz Y, Nakamura S, Heestand B, Liu W, et al. Small nucleoli are a cellular hallmark of longevity. Nat Commun. 2017;8:16083. 10.1038/ncomms16083. 28853436 10.1038/ncomms16083 PMC5582349

[58] Gentilella A, Morón-Duran FD, Fuentes P, Zweig-Rocha G, Riaño-Canalias F, Pelletier J, et al. Autogenous control of 5′TOP mRNA stability by 40S ribosomes. Mol Cell. 2017;67:55-70.e4. 10.1016/j.molcel.2017.06.005. 28673543 10.1016/j.molcel.2017.06.005 PMC5553558

[59] Delfarah A, Parrish S, Junge JA, Yang J, Seo F, Li S, et al. Inhibition of nucleotide synthesis promotes replicative senescence of human mammary epithelial cells. J Biol Chem. 2019;294:10564–78. 10.1074/jbc.RA118.005806. 31138644 10.1074/jbc.RA118.005806 PMC6615688

[60] Zwerschke W, Mazurek S, Stöckl P, Hütter E, Eigenbrodt E, Jansen-Dürr P. Metabolic analysis of senescent human fibroblasts reveals a role for AMP in cellular senescence. Biochem J. 2003;376:403–11. 10.1042/BJ20030816. 12943534 10.1042/BJ20030816 PMC1223775

[61] Passos JF, Nelson G, Wang C, Richter T, Simillion C, Proctor CJ, et al. Feedback between p21 and reactive oxygen production is necessary for cell senescence. Mol Syst Biol. 2010;6:347. 10.1038/msb.2010.5. 20160708 10.1038/msb.2010.5 PMC2835567

[62] Passos JF, Saretzki G, Ahmed S, Nelson G, Richter T, Peters H, et al. Mitochondrial dysfunction accounts for the stochastic heterogeneity in telomere-dependent senescence. PLoS Biol. 2007;5:e110. 10.1371/journal.pbio.0050110. 17472436 10.1371/journal.pbio.0050110 PMC1858712

[63] Dumollard R, Duchen M, Carroll J. The role of mitochondrial function in the oocyte and embryo. Curr Top Dev Biol. 2007;77:21–49. 10.1016/S0070-2153(06)77002-8. 17222699 10.1016/S0070-2153(06)77002-8

[64] Li YJ, Han Z, Ge L, Zhou CJ, Zhao YF, Wang DH, et al. C phycocyanin protects against low fertility by inhibiting reactive oxygen species in aging mice. Oncotarget. 2016;7:17393–409. 10.18632/oncotarget.8165. 27008700 10.18632/oncotarget.8165 PMC4951220

[65] Van Blerkom J. Mitochondrial function in the human oocyte and embryo and their role in developmental competence. Mitochondrion. 2011;11:797–813. 10.1016/j.mito.2010.09.012. 20933103 10.1016/j.mito.2010.09.012

[66] Van Blerkom J, Davis PW, Lee J. ATP content of human oocytes and developmental potential and outcome after in-vitro fertilization and embryo transfer. Hum Reprod. 1995;10:415–24. 10.1093/oxfordjournals.humrep.a135954. 7769073 10.1093/oxfordjournals.humrep.a135954

[67] Wiener-Megnazi Z, Vardi L, Lissak A, Shnizer S, Reznick AZ, Ishai D, et al. Oxidative stress indices in follicular fluid as measured by the thermochemiluminescence assay correlate with outcome parameters in in vitro fertilization. Fertil Steril. 2004;82(3):1171–6. 10.1016/j.fertnstert.2004.06.013. 15474091 10.1016/j.fertnstert.2004.06.013

[68] Wang C-H, Tsai T-F, Wei Y-H. Role of mitochondrial dysfunction and dysregulation of Ca^2+^ homeostasis in insulin insensitivity of mammalian cells. Ann N Y Acad Sci. 2015;1350:66–76. 10.1111/nyas.12838. 26214798 10.1111/nyas.12838

[69] Adams J. The proteasome: structure, function, and role in the cell. Cancer Treat Rev. 2003;29(1):3–9. 10.1016/s0305-7372(03)00081-1. 12738238 10.1016/s0305-7372(03)00081-1

[70] Karamysheva Z, Díaz-Martínez LA, Warrington R, Yu H. Graded requirement for the spliceosome in cell cycle progression. Cell Cycle. 2015;14:1873–83. 10.1080/15384101.2015.1039209. 25892155 10.1080/15384101.2015.1039209 PMC4614359

[71] Rousseau A, Bertolotti A. Regulation of proteasome assembly and activity in health and disease. Nat Rev Mol Cell Biol. 2018;19:697–712. 10.1038/s41580-018-0040-z. 30065390 10.1038/s41580-018-0040-z

[72] Hideshima T, Richardson P, Chauhan D, Palombella VJ, Elliott PJ, Adams J, et al. The proteasome inhibitor PS-341 inhibits growth, induces apoptosis, and overcomes drug resistance in human multiple myeloma cells. Cancer Res. 2001;61:3071–6. 11306489

[73] Shah SA, Potter MW, McDade TP, Ricciardi R, Perugini RA, Elliott PJ, et al. 26S proteasome inhibition induces apoptosis and limits growth of human pancreatic cancer. J Cell Biochem. 2001;82:110–22. 10.1002/jcb.1150. 11400168 10.1002/jcb.1150

[74] Kisselev AF, Goldberg AL. Proteasome inhibitors: from research tools to drug candidates. Chem Biol. 2001;8:739–58. 10.1016/s1074-5521(01)00056-4. 11514224 10.1016/s1074-5521(01)00056-4

[75] Suraweera A, Münch C, Hanssum A, Bertolotti A. Failure of amino acid homeostasis causes cell death following proteasome inhibition. Mol Cell. 2012;48:242–53. 10.1016/j.molcel.2012.08.003. 22959274 10.1016/j.molcel.2012.08.003 PMC3482661

[76] Will CL, Lührmann R. Spliceosome structure and function. Cold Spring Harb Perspect Biol. 2011;3:a003707. 10.1101/cshperspect.a003707. 21441581 10.1101/cshperspect.a003707 PMC3119917

[77] Ubaida-Mohien C, Lyashkov A, Gonzalez-Freire M, Tharakan R, Shardell M, Moaddel R, et al. Discovery proteomics in aging human skeletal muscle finds change in spliceosome, immunity, proteostasis and mitochondria. Elife. 2019;8:e49874. 10.7554/eLife.49874. 31642809 10.7554/eLife.49874 PMC6810669

[78] Feringa FM, Raaijmakers JA, Hadders MA, Vaarting C, Macurek L, Heitink L, et al. Persistent repair intermediates induce senescence. Nat Commun. 2018;9:3923. 10.1038/s41467-018-06308-9. 30254262 10.1038/s41467-018-06308-9 PMC6156224

[79] Pezone A, Olivieri F, Napoli MV, Procopio A, Avvedimento EV, Gabrielli A. Inflammation and DNA damage: cause, effect or both. Nat Rev Rheumatol. 2023;19:200–11. 10.1038/s41584-022-00905-1. 36750681 10.1038/s41584-022-00905-1

[80] Huggett SB, Stallings MC. Genetic architecture and molecular neuropathology of human cocaine addiction. J Neurosci. 2020;40:5300–13. 10.1523/JNEUROSCI.2879-19.2020. 32457073 10.1523/JNEUROSCI.2879-19.2020 PMC7329314

[81] Zhou P, Zhang J, Feng J, Wang G. Construction of an oxidative phosphorylation-related gene signature for predicting prognosis and identifying immune infiltration in osteosarcoma. Aging (Albany NY). 2024;16:5311–35. 10.18632/aging.205650. 38506898 10.18632/aging.205650 PMC11006489

[82] Haack TB, Madignier F, Herzer M, Lamantea E, Danhauser K, Invernizzi F, et al. Mutation screening of 75 candidate genes in 152 complex I deficiency cases identifies pathogenic variants in 16 genes including NDUFB9. J Med Genet. 2012;49:83–9. 10.1136/jmedgenet-2011-100577. 22200994 10.1136/jmedgenet-2011-100577

[83] Santidrian AF, Matsuno-Yagi A, Ritland M, Seo BB, LeBoeuf SE, Gay LJ, et al. Mitochondrial complex I activity and NAD+/NADH balance regulate breast cancer progression. J Clin Invest. 2013;123:1068–81. 10.1172/JCI64264. 23426180 10.1172/JCI64264 PMC3582128

[84] Tan AS, Baty JW, Berridge MV. The role of mitochondrial electron transport in tumorigenesis and metastasis. Biochim Biophys Acta. 2014;1840:1454–63. 10.1016/j.bbagen.2013.10.016. 24141138 10.1016/j.bbagen.2013.10.016

