# Supplementary material for: Multi-tissue analysis identifies mitochondrial genes in chicken aging-induced productivity decline
Source: J Anim Sci Biotechnol. 2026 Apr 22;17:75. doi: 10.1186/s40104-026-01392-0 (PMC13101107; doi:10.1186/s40104-026-01392-0)
Supplement: Supplementary file 2 — Additional file 2: Fig. S1. Integrated analysis of age-dependent gene expression and functional networks. Fig. S2. The result of MCODE analysis of the tissue-specific DEGs. Fig. S3. Line graph and results of cell staining for mitochondrial membrane potential, apoptosis, and SA-β-Gal staining. [file 40104_2026_1392_MOESM2_ESM.docx]

**Additional file 2**

**
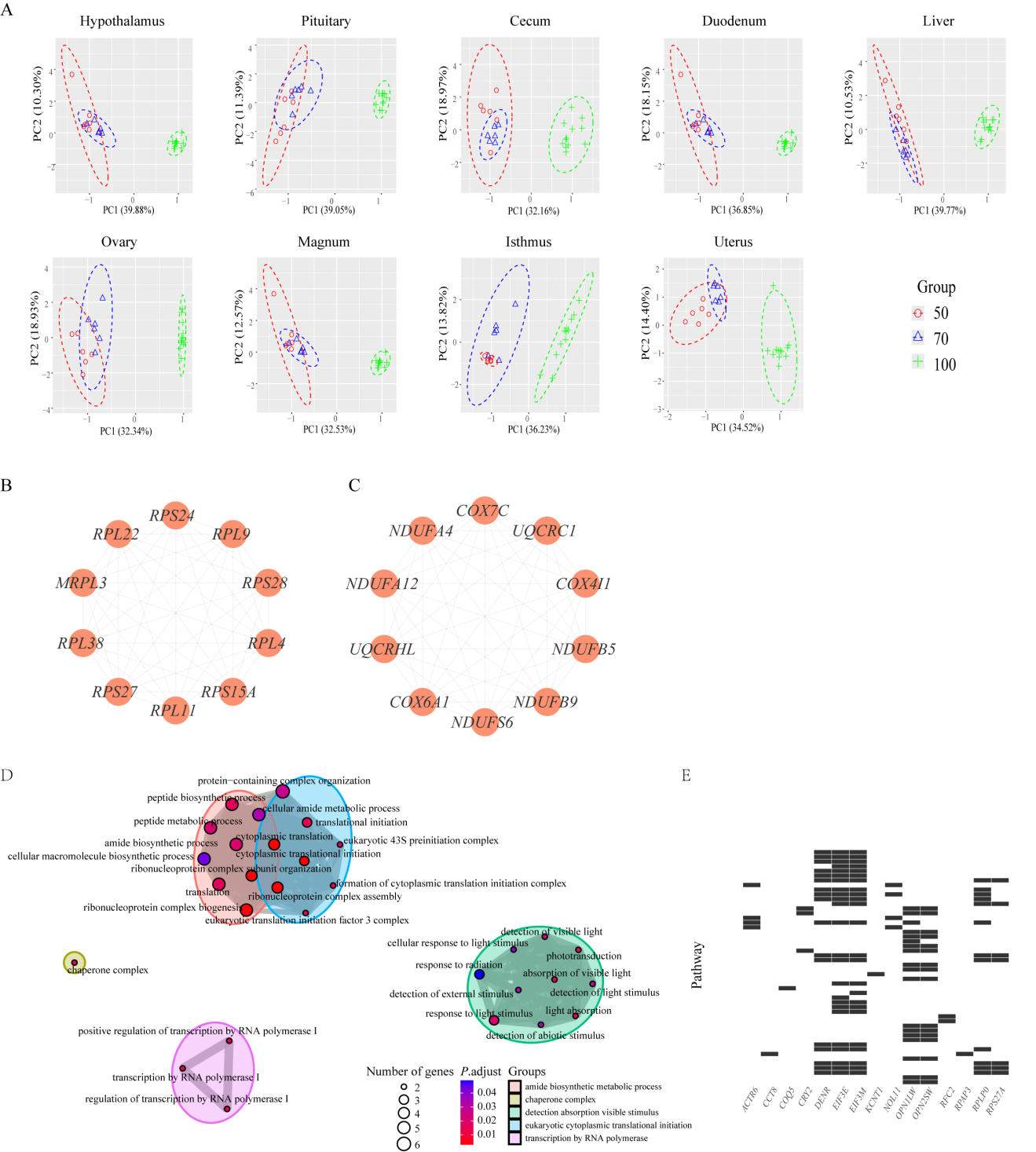
**

**Fig. S1** Integrated analysis of age-dependent gene expression and functional networks. **A** PCA plot of transcriptome data from 9 tissues at different weeks of age. 50 weeks of age is represented in red, 70 weeks of age is represented in blue, 100 weeks of age is represented in green. **B** The gene network of the ribosome. Ten hub genes labeled with orange. **C** The gene network of the mitochondria. Ten hub genes labeled with orange. **D** GO functional enrichment network analysis of the 87 highly conserved age-associated DEGs. **E** Heatmap of the DEGs across all GO terms. Black squares indicate gene enrichment in the corresponding term, while white squares indicate no enrichment.

**
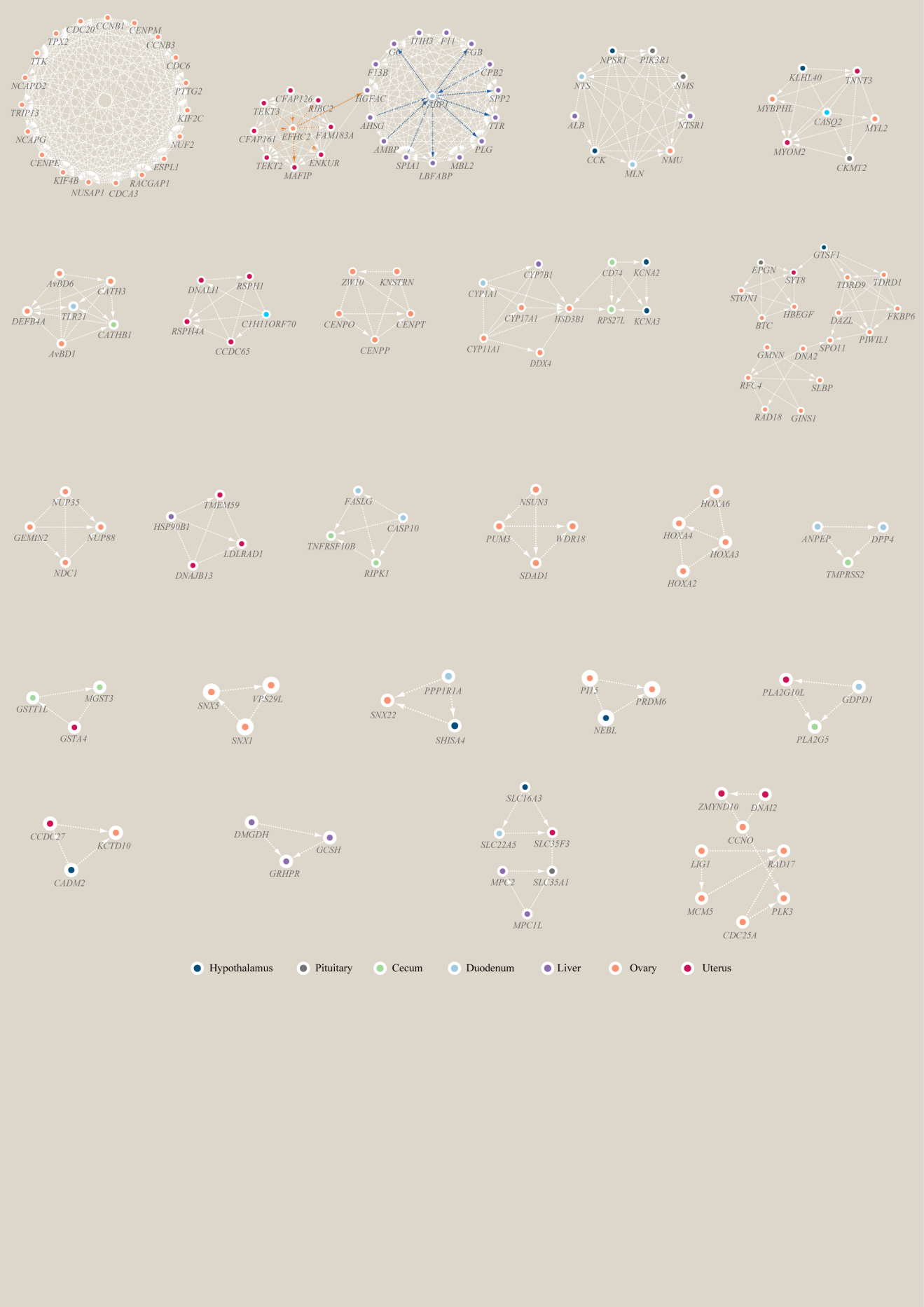
**

**Fig. S2** The result of MCODE analysis of the tissue-specific DEGs. The deep blue circles represent tissue-specific DEGs of the hypothalamus. The gray circles represent tissue-specific DEGs of the pituitary. The green circles represent tissue-specific DEGs of the cecum. The blue circles represent tissue-specific DEGs of the duodenum. The purple circles represent tissue-specific DEGs of the liver. The orange circles represent tissue-specific DEGs of the ovary. The red circles represent tissue-specific DEGs of the uterus. There are a total of 24 gene network modules.


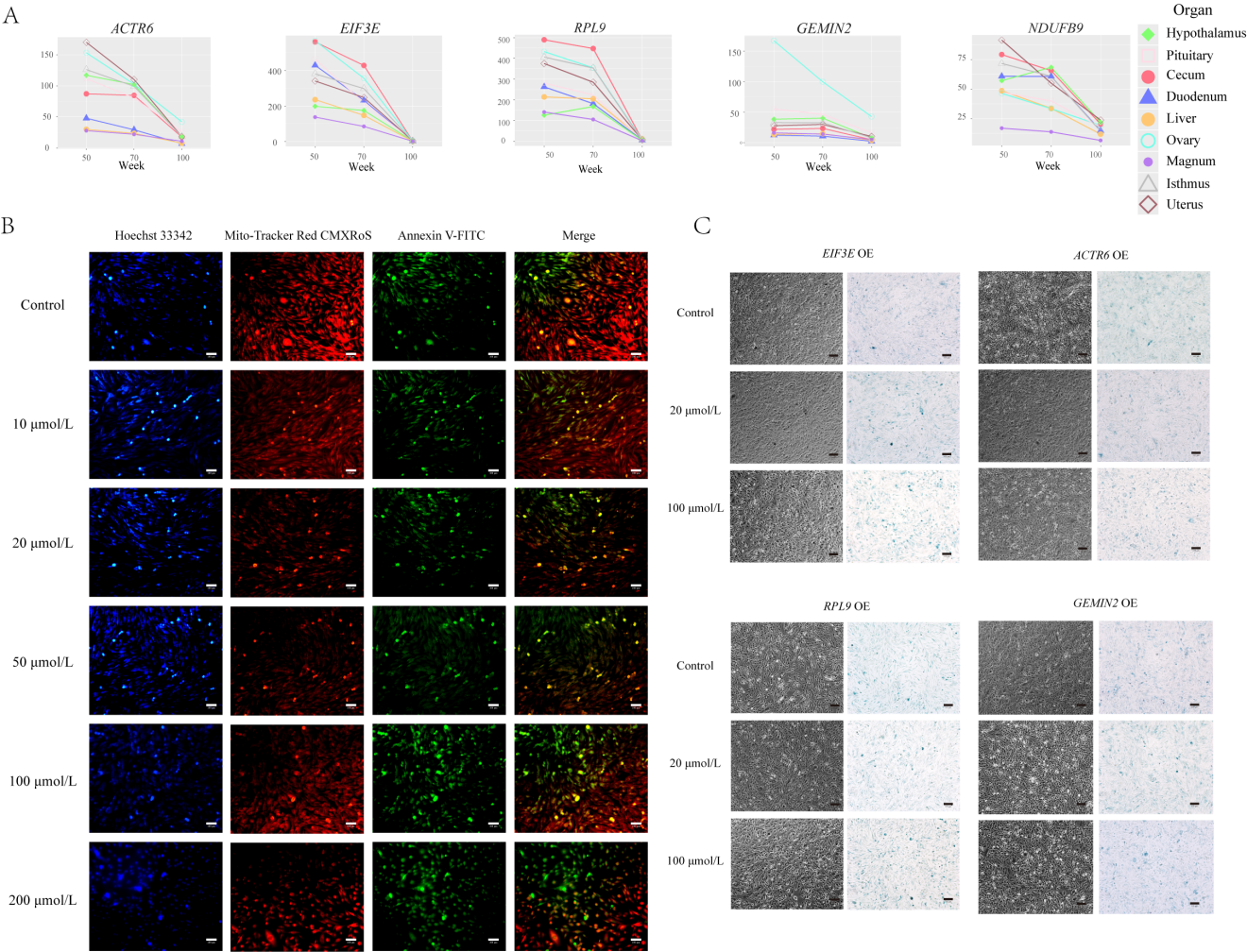


**Fig. S3** Line graph and results of cell staining for mitochondrial membrane potential, apoptosis, and SA-β-gal staining. **A** Line graph illustrating the changes in expression levels of *ACTR6*, *EIF3E*, *RPL9*, *GEMIN2*, *NDUFB9* across nine tissues. **B** Mitochondrial membrane potential and apoptosis detection. Green fluorescence indicates cellular apoptosis by Annexin V-FITC staining and red fluorescence reveals mitochondrial membrane potential by MitoTracker Red CMXRos staining. **C.** SA-β-gal staining results of *RPL9*, *ACTR6*, *EIF3E*, *GEMIN2* overexpressing DF-1 cells.
